# Relative Body Weight and Standardised Brightness-Mode Ultrasound Measurement of Subcutaneous Fat in Athletes: An International Multicentre Reliability Study, Under the Auspices of the IOC Medical Commission

**DOI:** 10.1007/s40279-019-01192-9

**Published:** 2019-09-30

**Authors:** Wolfram Müller, Alfred Fürhapter-Rieger, Helmut Ahammer, Timothy G. Lohman, Nanna L. Meyer, Luis B. Sardinha, Arthur D. Stewart, Ronald J. Maughan, Jorunn Sundgot-Borgen, Tom Müller, Margaret Harris, Nuwanee Kirihennedige, Joao P. Magalhaes, Xavier Melo, Wolfram Pirstinger, Alba Reguant-Closa, Vanessa Risoul-Salas, Timothy R. Ackland

**Affiliations:** 1 Biophysics, Medical University of Graz, Neue Stiftingtalstraße 6, 8010 Graz, Austria; 2 University of Arizona, Tucson, USA; 3 University of Colorado Colorado Springs, Colorado Springs, CO USA; 4 Faculdade Motricidade Humana, CIPER, Universidade Lisboa, Lisbon, Portugal; 5 Robert Gordon University, Aberdeen, UK; 6 School of Medicine, St Andrews University, St Andrews, UK; 7 NIH, The Norwegian School of Sport Sciences, Oslo, Norway; 8 University of Western Australia, Perth, Australia

## Abstract

**Introduction:**

Fat is a metabolic fuel, but excess body fat is ballast mass, and therefore, many elite athletes reduce body fat to dangerously low levels. Uncompressed subcutaneous adipose tissue (SAT) thickness measured by brightness-mode ultrasound (US) provides an estimate of body fat content.

**Methods:**

The accuracy for determining tissue borders is about 0.1–0.2 mm and reliability (experienced measurers) was within ± 1.4 mm (95% limit of agreement, LOA). We present here inter- and intra-measurer scores of three experienced US measurers from each of the centres C1 and C2, and of three novice measurers from each of the centres C3–C5. Each of the five centres measured 16 competitive adult athletes of national or international level, except for one centre where the number was 12. The following sports were included: artistic gymnastics, judo, pentathlon, power lifting, rowing, kayak, soccer, tennis, rugby, basketball, field hockey, water polo, volleyball, American football, triathlon, swimming, cycling, long-distance running, mid-distance running, hurdles, cross-country skiing, snowboarding, and ice hockey. SAT contour was detected semi-automatically: typically, 100 thicknesses of SAT at a given site (i.e., in a given image), with and without fibrous structures, were measured.

**Results:**

At SAT thickness sums D_I_ (of eight standardised sites) between 6.0 and 70.0 mm, the LOA of experienced measurers was 1.2 mm, and the intra-class correlation coefficient ICC was 0.998; novice measurers: 3.1 mm and 0.988. Intra-measurer differences were similar. The median D_I_ value of all 39 female participants was 51 mm (11% fibrous structures) compared to 17 mm (18%) in the 37 male participants.

**Discussion:**

D_I_ measurement accuracy and precision enables detection of fat mass changes of approximately 0.2 kg. Such reliability has not been reached with any other method. Although females’ median body mass index and mass index were lower than those of males, females’ median D_I_ was three times higher, and their percentage of fibrous structures was lower. The standardised US method provides a highly accurate and reliable tool for measuring SAT and thus changes in body fat, but training of measurers is important.

**Electronic supplementary material:**

The online version of this article (10.1007/s40279-019-01192-9) contains supplementary material, which is available to authorized users.

## Key Points

**Table:** 

Using the standardised B-mode ultrasound method, sums of subcutaneous adipose tissue (SAT) thicknesses (D) determined by experienced measurers at eight sites (on trunk, legs, and arms) can be determined with high accuracy and reliability: the 95% limit of agreement for experienced measurers (three in each of the two experienced study centres C1 and C2) was below 1.5 mm (embedded fibrous structures included: D_I_), and below 2.2 mm (fibrous structures excluded: D_E_). This enables monitoring changes of SAT mass in athletes (which forms the dominating part of total body fat) with an accuracy of about 0.2 kg. The median thickness measurement deviations at the individual eight sites were all below 0.2 mm. Measurement differences of novice measurers, after a 2-day course, were approximately three times larger.
This ultrasound method also allows to quantify the amount of fibrous structures (fasciae) embedded in the SAT: D_F_ = D_I_ − D_E_. The amount of this connective tissue was significantly lower in the 39 female elite athletes of various sports (median of 11%) when compared to the 37 male elite athletes (18%). Median SAT thickness sum D_I_ of the eight sites was three-times higher in the elite female athletes compared to their male counterparts (51 mm vs 17 mm).
In this group of elite athletes, there was no significant correlation between SAT and body mass index (BMI). The BMI is a measure of relative body mass, but not a useful tool to determine body fat. This holds also true for the mass index MI, but this improved measure for relative body mass considers the individual's leg length, which the BMI ignores. Differences (MI-BMI) were large in several cases and ranged from − 1.7 to + 1.3 kg m^−2^ (median BMI was 22.6 kg m^−2^), which supports the suggestion to include leg length (or sitting height) in all basic data sets of athletes and patients and thereby to assess body mass with respect to body dimensions in an improved way.

## Introduction

In 2013, a discussion paper dealing with the question of how to minimise the health risks to athletes who compete in weight-sensitive sports was presented by the Working Group on Body Composition, Health and Performance (under the auspices of the IOC Medical Commission) [1]. This working group also analysed advantages and shortcomings of widely used body fat assessment methods, including reference, laboratory, and field methods [2]. The authors stated: “…all of the techniques in common use have some inherent problems, whether in methodology, interpreting the data, or in the assumptions they make … Recent developments in ultrasound imaging have made possible accurate and reliable estimates of fat thickness in multiple sites of the body”.

Adipose tissue layer thicknesses can be measured by a standardised ultrasound (US) approach with an accuracy not reached by any other method [2–8]. This method can be used in all persons ranging from extremely lean to obese [7]. The method has been applied in various groups, including anorectic patients [9], obese persons [7], children [10], youth athletes [11], gymnasts and swimmers [6], and rowers [11]. However, these samples were small and comprehensive data of many sports are missing. Preliminary normative data for athletes and the general population have recently been suggested [12].

The accuracy for determining tissue borders is about 0.1–0.2 mm at 12–18 MHz probe frequency [3, 6] when the appropriate speed of sound for the given tissue is used for distance determination. A detailed description of factors determining the thickness measurement accuracy can be found in the Electronic Supplementary Material (ESM). High-frequency brightness-mode US (medical diagnostic ultrasound) is the only in vivo method that enables quantifying the fibrous structures embedded in the SAT. These structures, which are not composed of adipose cells, form a substantial part of the SAT that should not be ignored when assessing “fatness”. A preceding study [3] showed that the amount of these structures varied greatly, depending on both the measurement site and the person under investigation; in this studied group of 11 female football players (2nd league) and eight rhythmic gymnasts (national level), the fibrous structure median was 10%, and 50% of the values were between 6 and 17%. However, a structured analysis of the amounts of fibrous structures found in a group of male and female elite athletes of various sports has not been shown before the study presented here.

In several preceding publications [3–7], both the SAT thicknesses including the fibrous structures (indicated by the index “I”) and the SAT thicknesses excluding them (“E”) were measured. To measure both values is not only of interest for determining the amount of fibrous structures (i.e., the difference between these two thicknesses), but also for comparisons with other body fat measurement methods. Some techniques, such as imaging methods like magnetic resonance imaging (MRI) or computer tomography (CT), skinfolds, and cadaver studies, measure fat on the anatomical level, while others measure on the molecular level, for example the four-component model and the dual X-ray absorptiometry [2]. Accordingly, comparisons using either SAT thicknesses including or excluding the fibrous structures are of primary relevance. Such comparative studies (using the four-component-model, DXA, and MRI high-resolution scanning) are currently in progress in the centres that contributed to this study.

Inter- and intra-measurer reliability has been tested previously [4, 6, 7], but all these comparative measurements were performed by the experienced measurers of a single centre (referred to as “C1”), and test person samples were small and not representative for a wide range of sports. In 12 elite athletes (females: five gymnasts, one swimmer; males: 4 gymnasts, 2 swimmers), with sums of SAT thicknesses D_I_ (from the eight standardised measurement sites) ranging from D_I_ = 10 mm to D_I_ = 50 mm, 95% of scores between measurers were within ± 1.1 mm (from the mean of the three measurements) [6]. Similar inter-measurer reliability was reached when children were studied by two experienced measurers (of the centre C1) and additionally by a third experienced measurer (of a centre that did not participate in the multicentre study described here) [13]. In a group of 38 test persons (only two elite athletes, both sailors, were in this group) ranging from lean (D_I_ = 12 mm) to obese (D_I_ = 245 mm), 95% of repeated measurements of experienced measurers of centre C1 (intra-measurer reliability) were within ± 2.2 mm. In the sub-group ranging from D_I_ = 12–77 mm, 95% of values were within ± 1.4 mm, and in a second sub-group ranging from D_I_ = 44–245 mm, 95% were within ± 2.9 mm [7]. Typical differences between measurers (ΔD_I_) increased with increasing SAT thickness (d), but relative values (ΔD_I_/d) decreased [7].

As stated in the position statement of the IOC Working Group on Body Composition, Health and Performance [2], relative body weight determination in terms of the mass index (MI), which is a modified body mass index (BMI) considering the individual’s sitting height (or leg length), should be “included in all basic data sets of athletes and patients”. Considering leg length when determining relative body weight had been suggested by the Expert Committee on Physical Status of the World Health Organisation in 1995 [14].

This multicentre study aims to apply the recently developed and standardised B-mode ultrasound method for measuring SAT in a large group of elite male and female athletes (*N *= 76) of various sports to investigate the following topics:

1. Inter- and intra-measurer reliability obtained by 15 independently measuring experienced (6) and novice (9) measurers in research centres of five different countries.
2. Quantification of the SAT thickness sums and that of the fibrous structures embedded in the SAT (fasciae) in this group of elite athletes representing weight-sensitive and non-weight-sensitive sports, and to compare the elite male athletes (*N *= 37) to the elite female athletes (*N *= 39).
3. Comparison of the SAT values of such a mixed group of elite athletes to their BMI and analysis of the impact of the individual’s sitting height (which is inversely related to the leg length) on the assessment of body weight with respect to body dimensions.

## Methods

### Study Design, Participating Centres, and Measurers

SAT was measured with a recently standardised US technique [6] in five centres (C1–C5) in 36 weight-sensitive (w) and 40 non-weight-sensitive (nw) competitive athletes (national and international level). Each of the three measurers of a centre independently landmarked the eight sites on each of the athletes participating in his or her centre, and then captured and evaluated the US images. Thus, each athlete of a centre was measured three times; usually, marking, capturing the US images, and anthropometric data collection by the three measurers from each centre took place within 1 day (in the study design, the maximum time span had been fixed to 3 days). The marks on the skin were erased between the measurements of the three measurers (an erasable pen, e.g., an eyebrow pen, was used). The measurers did not have access to the results obtained by the other (two) measurers of their individual centre.

In addition, a second US image was captured in a sub-set of 56 athletes; all measurers assessed the intra-measurer participants of their centre twice: one image per site was taken, and then, this was repeated for the second image. After the first image had been captured, the US gel was removed from the skin and from the probe, and a new thick layer of gel was loaded onto the probe before the second image was taken.

Intra-measurer reliability of US image capturing and thickness evaluation was determined that way (in contrast to the inter-measurer reliability that included all three components: marking, image capturing, and thickness evaluation). Again, the measurers did not have access to the results obtained by the other measurers.

Measurers with different experiences were involved: those from centres C1–C2 were very experienced in handling the US systems (which belonged to their laboratories), and the other three centres (C3–C5) had limited experience in US imaging (and used a borrowed US system), apart from a 2-day course on US measurement and evaluation technique, followed by a supervised measurement series in five test persons.

All participants were informed about the aims and methods and gave their written consent for anonymous use of their personal data. The local ethics committees approved data collection at the five centres (in alphabetical order): Aberdeen: Robert Gordon University, UK (12-413); Colorado Springs: University of Colorado, USA (IRB14-211); Graz: Medical University of Graz, Austria (20-295 ex 08/09); Lisbon: University of Lisbon, Portugal (CEFMH, 16/2016); Perth: University of Western Australia, Australia (RA/4/1/6084). All centres declare that the study was performed in accordance with the standards of ethics outlined in the Declaration of Helsinki. The local Human Research Ethics bodies at each of the test centres follow these principles and guidelines for conducting research with human subjects.

### Participants and Groups

Each of the five centres measured eight competitive adult athletes in w sports and eight in nw sports, except at C3 where the number of nw athletes was four. All athletes were in training and participated in national or international competitions. They had not undertaken strenuous exercise during the previous 48 h and reported to be normally hydrated. Inclusion criteria were as follows. Age range was 17–35 years and participants were selected from a pre-defined list of sports. Weight-sensitive sports were defined in Ackland et al. [2]; they can be summarised in three groups: aesthetic sports, weight-class sports, and gravitational sports (in which mass restricts performance due to gravitational reasons). The study design stated that all centres should capture two US images of at least eight athletes (one athlete whose weight was above his weight-class limit was eliminated; therefore, only 7 w athletes of C3 were included).

Athletes from the following sports were included: C1: w: artistic gymnastics; nw: swimming, ice hockey; C2: w: triathlon, mid-distance running, hurdles, judo, pentathlon; nw: swimming, tennis; basketball; C3: w: cross-country skiing, snowboarding, road race cycling, power lifting; nw: swimming, field hockey, soccer, rugby, open class rowing; C4: w: triathlon, mid-long-distance running; nw: swimming, water polo, kayak. C5: w: cycling, running; nw: soccer, American football, volleyball.

All 76 participants (37 males: 21 w, 16 nw; 39 females: 15 w, 24 nw) were investigated three times (by the three measurers at each centre; study part A).

For the inter-measurer analyses, athletes with D_I_ > 70 mm were excluded (exclusion of physically not well-trained athletes). This exclusion criterion resulted in a total number of 65 athletes, 30 from the expert centres C1 and C2, and 35 from the novices centres C3–C5.

A sub-group of 56 (27 w, 29 nw) participants had a second US image captured at each marked site (study part B): The five centres contributed the following numbers of athletes: C1: 8 (4 w, 4 nw); C2: 16 (8, 8), C3: 7 (1, 6), C4: 9 (6, 3), and C5: 16 (8, 8). Thus, the expert group (C1 and C2) consisted of 24 athletes (12 w, 12 nw), and the novices group of 32 athletes (15, 17). The criterion D_I_ > 70 mm was also applied here, resulting in a total of 47 athletes participating in study part B (intra-measurer reliability).

Results obtained with all 76 athletes are presented in Fig. 2. In this context, the index “mean” refers to the means of the three measurements (three measurers measured each athlete of their centre, i.e., all athletes were measured three times). In Figs. 3 and 4, only athletes with sums of SAT thicknesses below D_I,mean_ = 70 mm were used; that is, only athletes with low or moderate amounts of body fat. This reduced the number of athletes of the experienced centres (C1 and C2) from 32 to 30 (i.e., 90 measurements remained). The median BMI for this sub-sample was 22.2 kg m^−2^, ranging from 19.2 to 27.9 kg m^−2^, and the IQR was 2.1 kg m^−2^. The number of athlete of the three novice groups together (C3:12 athletes, C4:16, and C5:16) was reduced by this limit from 44 to 35 (i.e., 105 measurements).

The intra-measurer study (Fig. 5) included 56 participants (C1:8 athletes, C2:16, C3:7, C4:9, C5:16); the athletes of each centre were measured twice by each of the three measurers of this centre.

### Anthropometry

Anthropometric measurements included body mass *m*, stature *h*, sitting height *s*, and leg length *l* (measured from the floor to the anterior superior iliac spine, ASIS). Two measures for relative body weight, the body mass index BMI = *m*/*h*^2^ and the mass index ${\text{MI}}_{1} = 0.53\;{{m}}/({{hs}})$, were calculated [15–17]. For derivation of the MI_1_ equation, see ESM.

### Site Marking and US Image Capture

The standardised US method has been described recently [6]. The external oblique site (EO) used in this study can cause measurement problems in obese persons and was, therefore, replaced in following measurement series by the lateral thigh site (LT), which is the thickest SAT fat depot in most women [7].

**Fig. 1:**
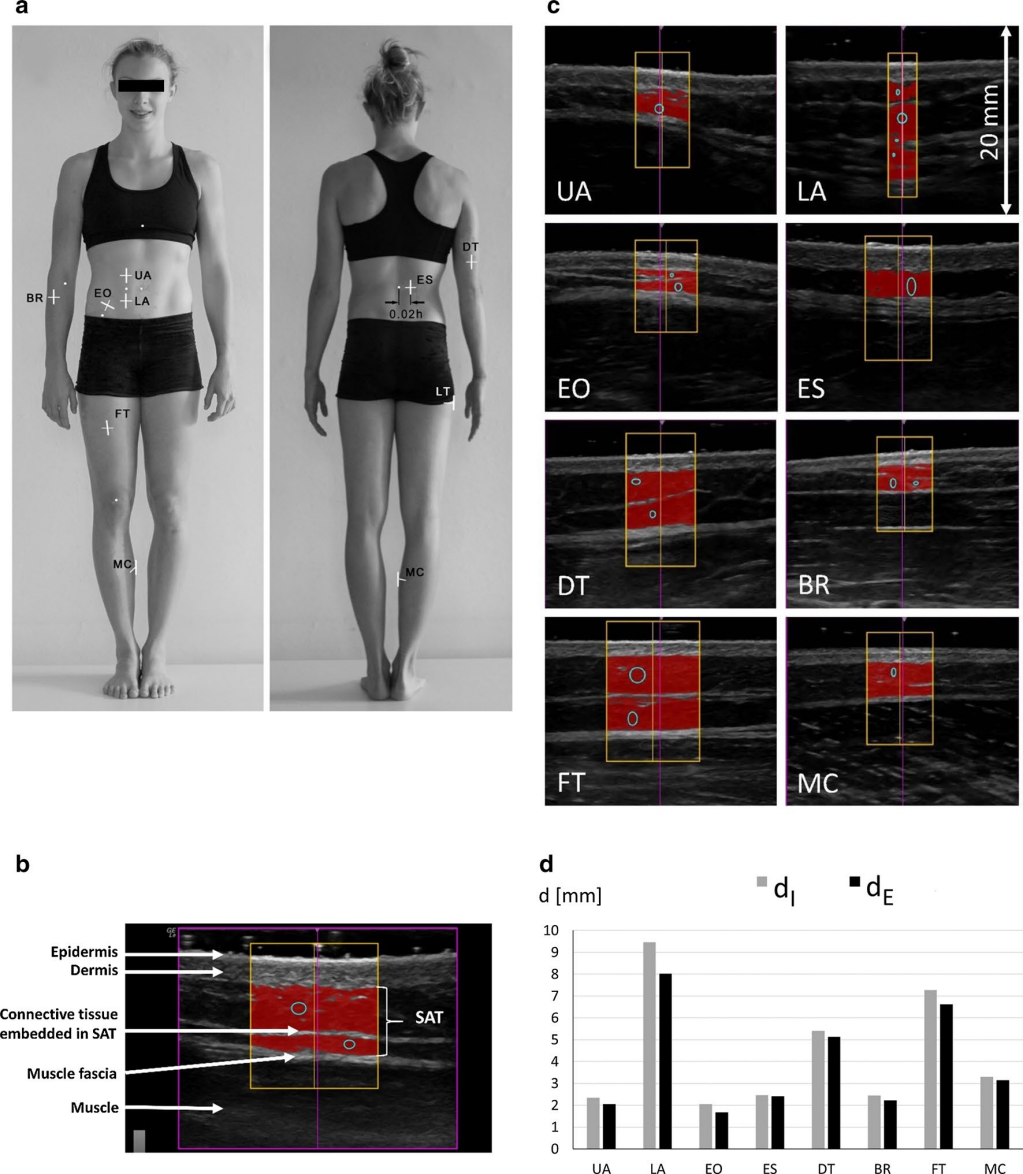
Standardised ultrasound (US) measurement of subcutaneous adipose tissue (SAT). **a** US sites. *UA* upper abdomen, *LA* lower abdomen, *EO* external oblique, *ES* erector spinae, *DT* distal triceps, *BR* brachioradialis, *FT* front thigh, and *MC* medial calf. Instead of the now standard lateral thigh (LT) site, the EO site was in use when this study was performed. For a detailed description of site marking, see [6, 7]. **b** B-mode US image of SAT. All eight sites show the same structure: the black band on top represents the thick gel layer (to avoid tissue compression), followed by the structures described in the figure. The amount of embedded fibrous structures (connective tissue) varies between individuals and from site to site; this holds also true for the skin thickness. **c** Evaluated US image series. Red areas represent the SAT detected by the semi-automatic contour detection algorithm [3, 6]; the ellipses indicate the regions where the algorithm started out for SAT contour detection. In this image series, the evaluation software determined between 48 (LA) to 155 (FT) thickness values within the rectangular ROI. **d** Survey of results: SAT patterning. SAT thicknesses with the embedded fibrous structures included (d_I_), and excluded (d_E_). The sum of the eight d values is termed D. In this participant, D_I_ was 34.8 mm, D_E_ was 31.3 mm, and about 10% of the mean depth comprised fibrous structures, while 90% was lipid, water, and adipose cell structures. BMI of this athlete was 22.0 kg m^−2^, and MI was 21.7 kg m^−2^

All eight US sites (Fig. 1a) were marked on the right side of the body. US images were captured with the participants lying in a defined supine, prone, or rotated position [6]. Compression of SAT was avoided using a thick layer of US gel [3, 6] between the US probe and the skin (the dark gel band can be seen on top of the US images, Fig. 1b, c). The probe was always held perpendicularly to the skin at the given site, with the centre of the probe positioned exactly above the marking.

The following brightness-mode (B-mode) US systems with linear probes operated between 12 and 18 MHz were used: C1 and C5: GE-logiq-e, probe-L8-18i, and probe 12L [General Electric, country of origin: China]; C2: Esaote Mylab One, 13 MHz [Esaote, Italy]; C3 and C4: Telemed, Echo-Blaster 128EXT-1Z-REV:C, probe-HL9.0/40/128Z-4, software Echo-WaveII-v3.2.0 [UAB Telmed, Lithuania].

### Contour Detection and SAT Thickness Measurements

Ultrasound images were evaluated interactively using an evaluation software (Rotosport, Austria; rotosport.at) for semi-automatic evaluations of SAT thicknesses. Sound speed was set to 1450 m s^−1^ for distance determination in fat [3, 18–22]. At each site, the software detects the SAT segments between the lower border of the skin and the upper border of the muscle fascia (for example, see Fig. 1b, c). The algorithm measures many thicknesses (typically 50–200) within the region of interest (ROI). The mean of these thickness values is termed *d* and represents the SAT thickness at the site. Sums of the eight sites D_I_ = d_I,1_ + ···+ d_I_,_8_ (including embedded structures) and sums D_E_ = d_E,1_ + ···+ d_E_,_8_ (fibrous structures excluded) were calculated. Tissue segmentation was controlled visually and could be improved, if necessary, by changing the algorithm parameters. Figure 1c shows a series of eight thickness measurements for a single participant. The centre line in the US image corresponds to the central US beam; the centre of the probe was held exactly above the marking of the site. The ROI was usually set symmetrically to the centre line. A final visual control made sure that the algorithm detected the SAT layer correctly. Images in Fig. 1c correspond to the SAT patterning shown in Fig. 1d.

### Statistics

SPSS (v23) software was used. Because data were not normally distributed in all sub-sets (Shapiro–Wilk test), Mann–Whitney-U test was applied for comparisons between male and female participants and between novice and experienced measurers. Correlation was tested with Spearman’s rank-order correlation coefficients ρ for: D_I_ and BMI, D_I_ and MI, D_I_ and D_I,mean_, D_E_ and D_E,mean_, D_F, %_ and D_I,mean_, D_I,meanB_ and D_I,meanA_, and D_I,AB_ and D_I,meanAB_. Limit of agreement (LOA ≈ 1.96∙SD) [23], and intra-class correlation (according to McGraw and Wong convention) [24] were computed to quantify differences between measurers: ICC(A,1)—two-way mixed, single score, absolute agreement or ICC(A,k)—two-way mixed, average score, absolute agreement. In addition, coefficients of determination *R*^2^ and standard errors of estimates SEE were computed. Variables and indices: D (sum of SAT thicknesses), I (fibrous structures are included), E (fibrous structures excluded), F, % (fibrous structures in  %), M1 (measurer 1), M2 (measurer 2), and M3 (measurer 3). A and B denote measurement series A (inter-measurer tests) and B (intra-measurer tests). Note: Not to be mixed up with the “A” used in the ICC convention (McGraw and Wong). Box plots, medians, first and third quartiles (Q), and interquartile ranges (IQR) were used to characterise distributions.

## Results

The survey plot (Fig. 1d) sketches both the SAT patterning with fibrous structures included in the SAT thickness (d_I_) or excluded (d_E_). The difference d_F_ = d_I_–d_E_ is the mean thickness of the embedded fibrous structures. The tissue layer thickness (d) at a given site is the mean of many (typically about 100) thickness measurements within the ROI, which is usually set symmetrically to the centre line (Fig. 1b, c). In the image series shown in Fig. 1c, the number of thickness measurements for determining the mean thicknesses d_I_, d_E_, and d_F_ at these eight sites ranged from 48 to 155.

The sum of the eight d_I_ values is termed D_I_ (sums of d_E_ values: D_E_). Figure 1c, d shows the example of a typical female gymnast (BMI: 22.0 kg m^−2^; MI: 21.7 kg m^−2^): D_I_ was 34.8 mm, D_E_ was 31.3 mm, and fibrous structures D_F_ = D_I_–D_E_ amounted to 3.5 mm (i.e., about 10% of total SAT thickness). When this multicentre study was performed, the site EO was in use instead of LT.

In all participants (*N *= 76) of the five study centres (C1–C5), SAT thicknesses were measured three times (by the three measurers of each centre) at the eight standardised sites (Fig. 1) [6, 7]. These 1824 ultrasound (US) measurements of thickness values d, form the core data set for the inter-measurer reliability study (measurement series A). In addition, in a sub-set of 56 participants, US imaging and SAT thickness evaluation were performed twice. This second measurement series is termed B. These 1344 repeated US measurements together with the corresponding 1344 measurements of series A form the data set for the intra-measurer reliability study.

BMI was significantly lower (*p *= 0.048) according to Mann–Whitney-U in females (median was 22.1 kg m^−2^) than in males (23.1 kg m^−2^). This also holds true (*p *< 0.01) for the improved measure for relative body mass MI_1_ (the MI_1_ was not determined in centre C4, thus *N *= 60). The differences MI-BMI ranged from − 1.7 to + 1.3 kg m^−2^. All data of individual athletes (Table A1) and of all sub-groups (Table A2) are listed in the ESM.

**Fig. 2:**
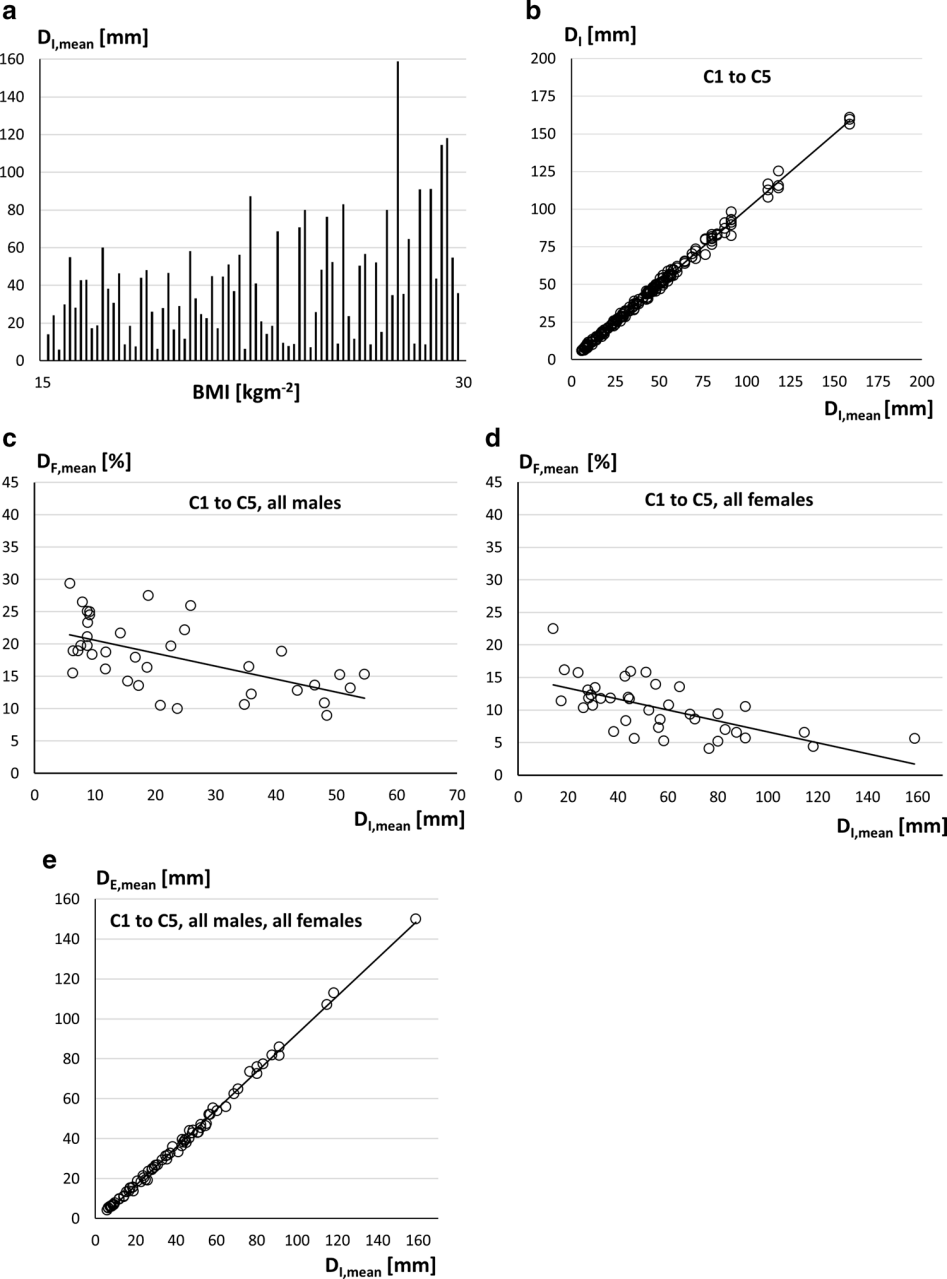
Subcutaneous adipose tissue (SAT) and embedded fibrous structures (F). In each of the five measurement centres (C1–C5), eight weight-sensitive (w) and eight non-weight-sensitive (nw) athletes (except for C3: only four in group w) were measured three times (by the three measurers of each centre). Each of the three measurers of each centre separately marked the eight sites of all athletes of his centre, captured the according US images, and evaluated them on his own. D_I_ and D_E_ indicate sums of SAT thicknesses from the eight standardised ultrasound sites with fibrous structures included or excluded, respectively. The index “mean” indicates that means of the three measurers were used. Values of all 76 athletes (37 male and 39 female participants; 12 participants of centre C3, 16 of each other centre). The difference D_F,mean_ = D_I,mean_ − D_E,mean_ represents the sum of embedded fibrous structures, and D_F, %_ = 100·D_F,mean_/D_I,mean_. For data of individuals, see Table A1 (Electronic Supplementary Material). **a** D_I,mean_ values and BMI. BMI (ranging from 17.9 to 29.0 kg m^−2^) did not correlate with D_I,mean_ (*R*^2^ = 0.130, Spearman’s *ρ *= 0.286). For individual values, see Electronic Supplementary Material, Table 1B. D_I_ ranged from 5.8 to 54.7 mm in males, and from 14.0 to 158.8 mm in females. Data of the groups are presented in Table 1 and in the Electronic Supplementary Material (Table A1 and Table A2). **b** Measurers’ individual D_I_ results. 15 measurers (three from each centre) assessed all athletes at their centres. The coefficient of determination was *R*^2^ = 0.997, *ρ *= 0.997, and SEE = 1.76 mm. **c** Percentage of embedded fibrous structures in male participants: D_F, %_ = 100 · D_F,mean_/D_I,mean_. The males’ median percentage was 18.3%, *R*^2^ = 0.346, and *ρ *= − 0.601. **d** Percentage of embedded fibrous structures in female participants: D_F, %_ = 100 · D_F,mean_/D_I,mean_. The females’ median percentage was substantially lower when compared to that of males: 10.5%, *R*^2^ = 0.414, and *ρ *= −0.667. **e** Correlation of D_I,mean_ and D_E,mean_: *R*^2^ = 0.997, and SEE = 1.5 mm

The means of the three SAT thickness measurements of each of the 76 athletes are shown in Fig. 2a. The athletes are ordered according to increasing BMI, which ranged from 17.9 to 29.0 kg m^−2^. There was no correlation between subcutaneous fat (D_I_) and BMI (*R*^2^ = 0.130, *ρ *= 0.286) or MI (*R*^2^ = 0.086, SEE = 29.4 mm, and *ρ *= 0.149).

The individual results for D_I_ of the three measurers of all centres C1-C5 (experienced and novice measurers together) are shown in Fig. 2b: *R*^2^ was 0.997, SEE = 1.8 mm, and *ρ *= 0.997. The respective values for D_E_ (not shown in a figure) were: *R*^2^ = 0.994, SEE = 2.1 mm, and *ρ *= 0.996.

In the group of solely weight-sensitive (w) sports, male participants had a median of D_I_ = 9.5 mm (IQR = 16.9 mm, minimum: 5.8 mm, maximum: 55.0 mm), and female participants had a median D_I_ = 33.1 (16.3, 14.0, 55.0 mm). In the non-weight-sensitive (nw) sports group, the male participants’ median was 23.1 (26.4, 8.8, 54.7 mm), and female participants’ median was 66.7 (33.2, 18.5, 158.8 mm).

With regard to the embedded fibrous structures in the SAT (Fig. 2c, d), for all 76 athletes, median D_F_ was 4.0 mm, ranging from 1.0 to 9.5 mm, and the median percentage of embedded fibrous structures was D_F,%_ = 13.3%, ranging from 4.0 to 29.3%. For all male participants (Fig. 2c), median D_F,%_ = 18.3%, ranging from 8.9 to 29.3%. In female participants (Fig. 2d), 10.5% fibrous structures were contained in the SAT, ranging from 4.0 to 22.5%. The percentage of fibrous structures tended to decrease in both men (*R*^2^ = 0.346) and women (*R*^2^ = 0.414) with increasing D_I,mean_. The means D_I,mean_, D_E,mean_, D_F,mean_, and D_F, %_ differed significantly between sexes (*p *< 0.001; groups: All_m_, All_f_).

D_I,mean_ values are closely correlated with D_E,mean_ (fibrous structures excluded). Figure 2e shows the comparison of D_I,mean_ and D_E,mean_ for all 76 athletes (means of the three measurers’ values): the coefficient of determination *R*^2^ was 0.997, SEE was 1.5 mm, and the slope of the regression line was 0.949. The slope was lower in the male (0.883) than in the female group (0.960), which mirrors the higher percentage of embedded fibrous structures in male athletes.

A comparison of male and female participants within the same D_I_ interval ranging from 14.0 to 60.0 mm (i.e., from the lowest female to the highest male value) also resulted in a significantly (*p *< 0.01) higher percentage of fibrous structures (D_F, %_) in male (median: 15%) than in female participants (12%), although the D_I,mean_, D_E,mean_, and D_F,mean_ values did not differ significantly in these sub-groups (*p *= 0.14, *p *= 0.10, *p *= 0.32, respectively).

### Inter-measurer Reliability in SAT Thickness

**Fig. 3:**
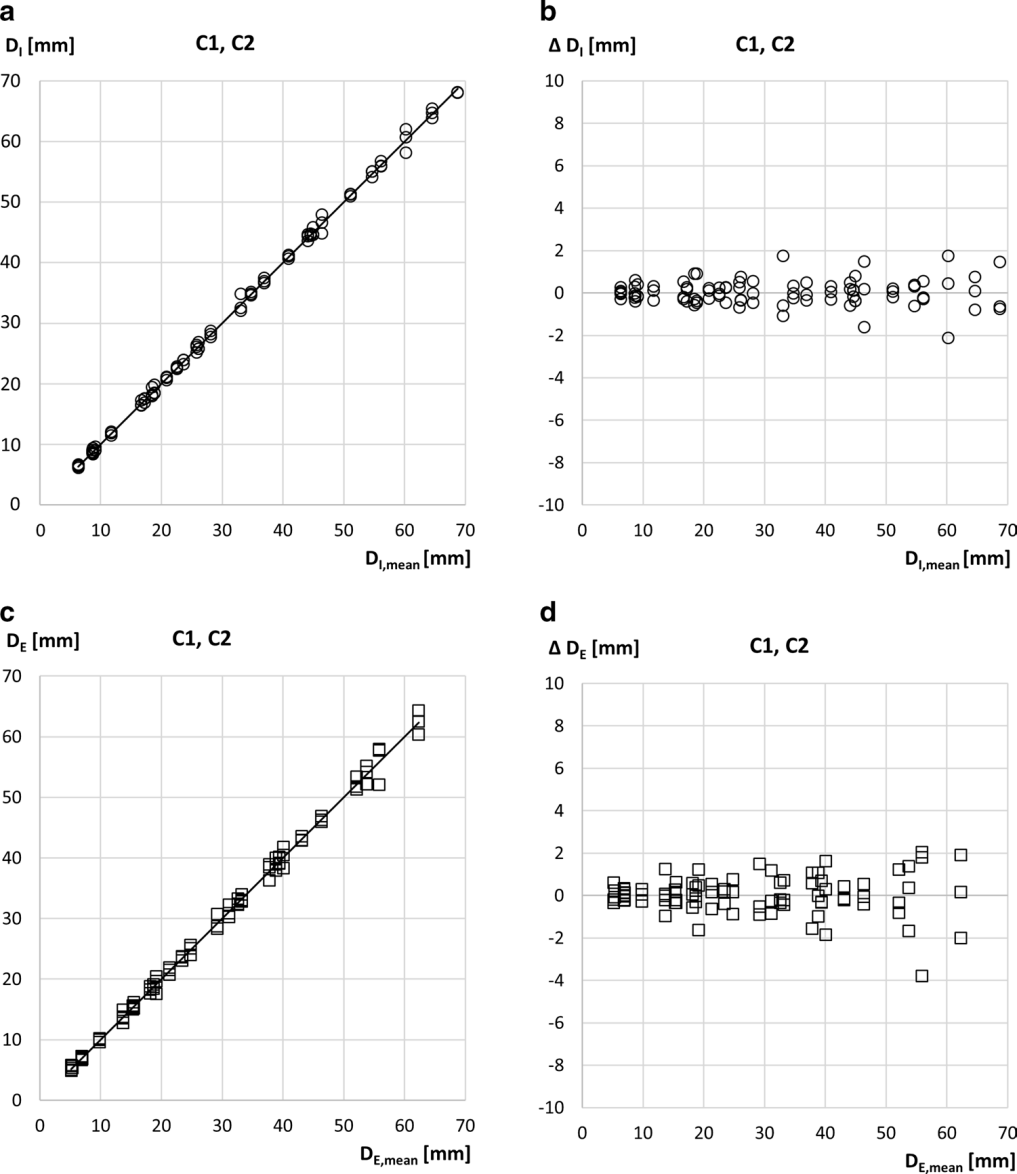
Inter-measurer comparisons at experienced centres (C1–C2). Results of the three experienced measurers of each of the two centres. This sub-group of athletes, with an upper limit of D_I,mean_ = 70 mm (i.e., physically trained athletes with low or moderate amounts of body fat [12]), included 16 male and 14 female participants. D_I_ is the sum of eight SAT thicknesses d_I_ (fibrous structures included). D_E_ is the sum of eight SAT thicknesses d_E_ (fibrous structures excluded). **a** D_I_ values obtained by three experienced measurers in each athlete. ICC(A1) = 0.998, SEE = 0.6 mm, and *ρ *= 0.998. **b** D_I_ deviations of the three experienced measurers from their mean. SD = 0.60 mm; limit of agreement (LOA) = 1.2 mm. **c** D_E_ values measured by three experienced measurers in each athlete. ICC(A1) = 0.996, SEE = 0.9 mm, and *ρ *= 0.996. **d** D_E_ deviations of the three experienced measurers from their mean. SD = 0.9 mm; LOA = 1.7 mm

Inter-measurer reliability among experienced examiners (C1–C2) is compared in Fig. 3a–d. The sub-group consisted of 30 athletes (each measured three times) with an upper limit of D_I,mean_ = 70 mm. Median BMI for this sub-sample was 22.2 kg m^−2^, and ranged from 19.2 to 27.9 kg m^−2^, IQR = 2.1 kg m^−2^.

For the D_I_ data of Fig. 3a, *R*^2^ was 0.999, SEE = 0.6 mm, *ρ *= 0.998, and ICC(A,1) = 0.998. For the D_E_ data (Fig. 3c), *R*^2^ was 0.997, SEE = 0.9 mm, *ρ *= 0.996, and ICC(A,1) = 0.996. Figure 3b shows the differences of the three measurers’ individual D_I_ differences from their mean; SD = 0.6 mm, and the limit of agreement LOA = 1.2 mm. Figure 3d shows the results for D_E_: SD = 0.9 mm, and LOA = 1.7 mm.

**Fig. 4:**
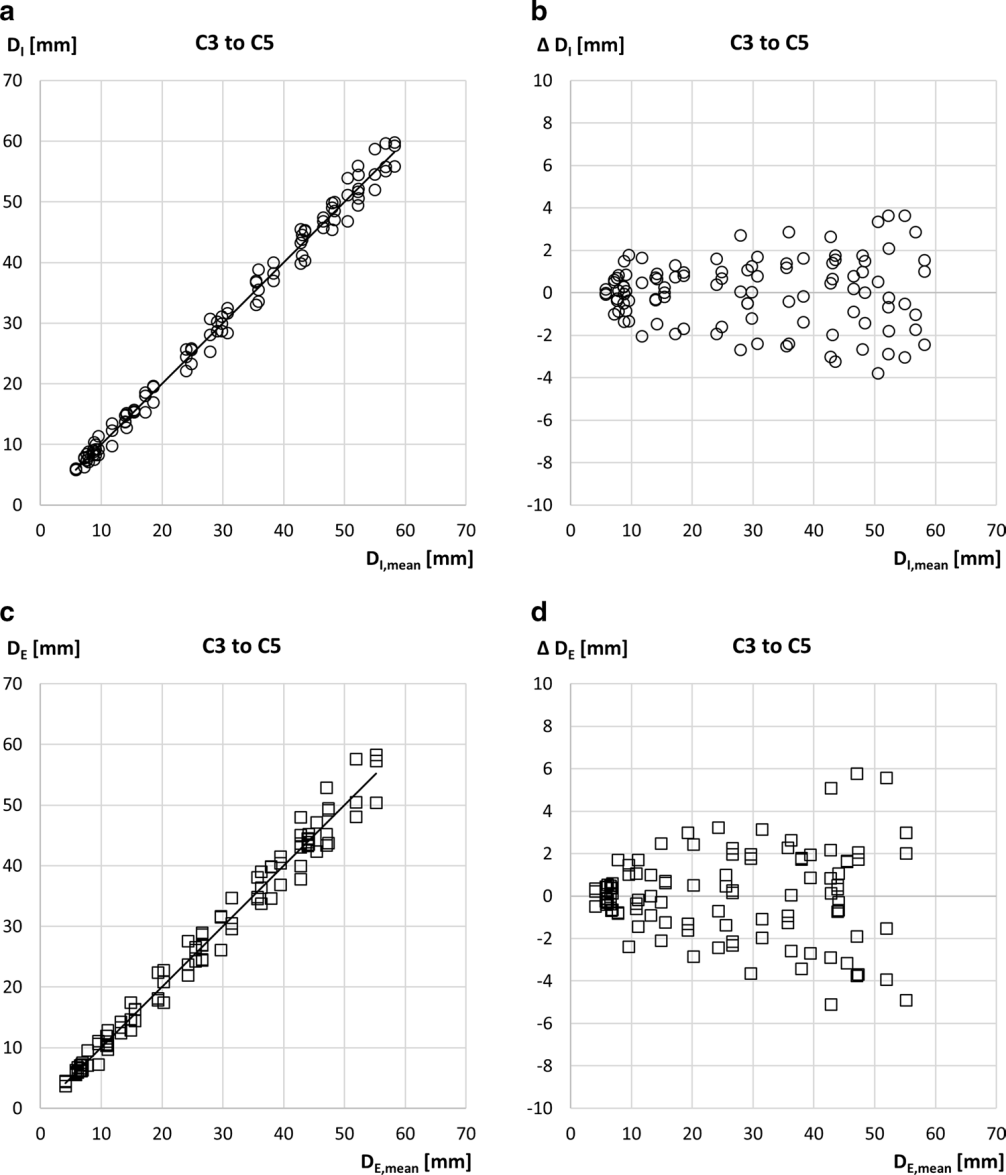
Inter-measurer comparisons at novice centres (C3–C5). This sub-group of athletes, with an upper limit of D_I,mean_ = 70 mm (i.e., physically trained athletes with low or moderate amounts of body fat), included 21 male and 14 female participants. D_I_ is the sum of eight SAT thicknesses d_I_ (fibrous structures included). D_E_ is the sum of eight SAT thicknesses d_E_ (fibrous structures excluded). **a** D_I_ values obtained by three novice measurers in each athlete. ICC(A1) = 0.988, SEE = 1.6 mm, and *ρ *= 0.993. **b** D_I_ deviations of the three novice measurers from their mean. SD = 1.6 mm; limit of agreement (LOA) = 3.1 mm. **c** D_E_ values measured by three novice measurers in each athlete. ICC(A1) = 0.977, SEE = 2.1 mm, and *ρ *= 0.989. **d** D_E_ deviations of the three novice measurers from their mean. SD = 2.0 mm; LOA = 4.0 mm

Figure 4a–d shows the comparisons of novice measurers from centres C3–C5. The sub-group consisted of 21 male and 14 female athletes (105 data points) with an upper limit of D_I,mean_ = 70 mm. The median BMI in this sub-group was 22.1 kg m^−2^ and ranged from 17.9 to 29.0 kg m^−2^, IQR = 3.4 kg m^−2^.

For the D_I_ data of Fig. 4a, *R*^2^ was 0.992, SEE = 1.6 mm, *ρ *= 0.993, and ICC(A,1) = 0.988; and for the D_E_ data of Fig. 4c, *R*^2^ was 0.984, SEE = 2.0 mm, *ρ *= 0.989, and ICC(A,1) = 0.977. Figure 4b shows the differences of the three measurers’ individual D_I_ differences from their mean; SD = 1.6 mm, and LOA = 3.1 mm. Figure 4d shows the results for D_E_: SD = 2.0 mm, and LOA = 4.0 mm.

Compared to experienced measurers, novices had significantly higher measurement deviations ΔD_I_ and ΔD_E_ (*p *< 0.001).

### Intra-measurer Reliability in SAT Thickness

**Fig. 5:**
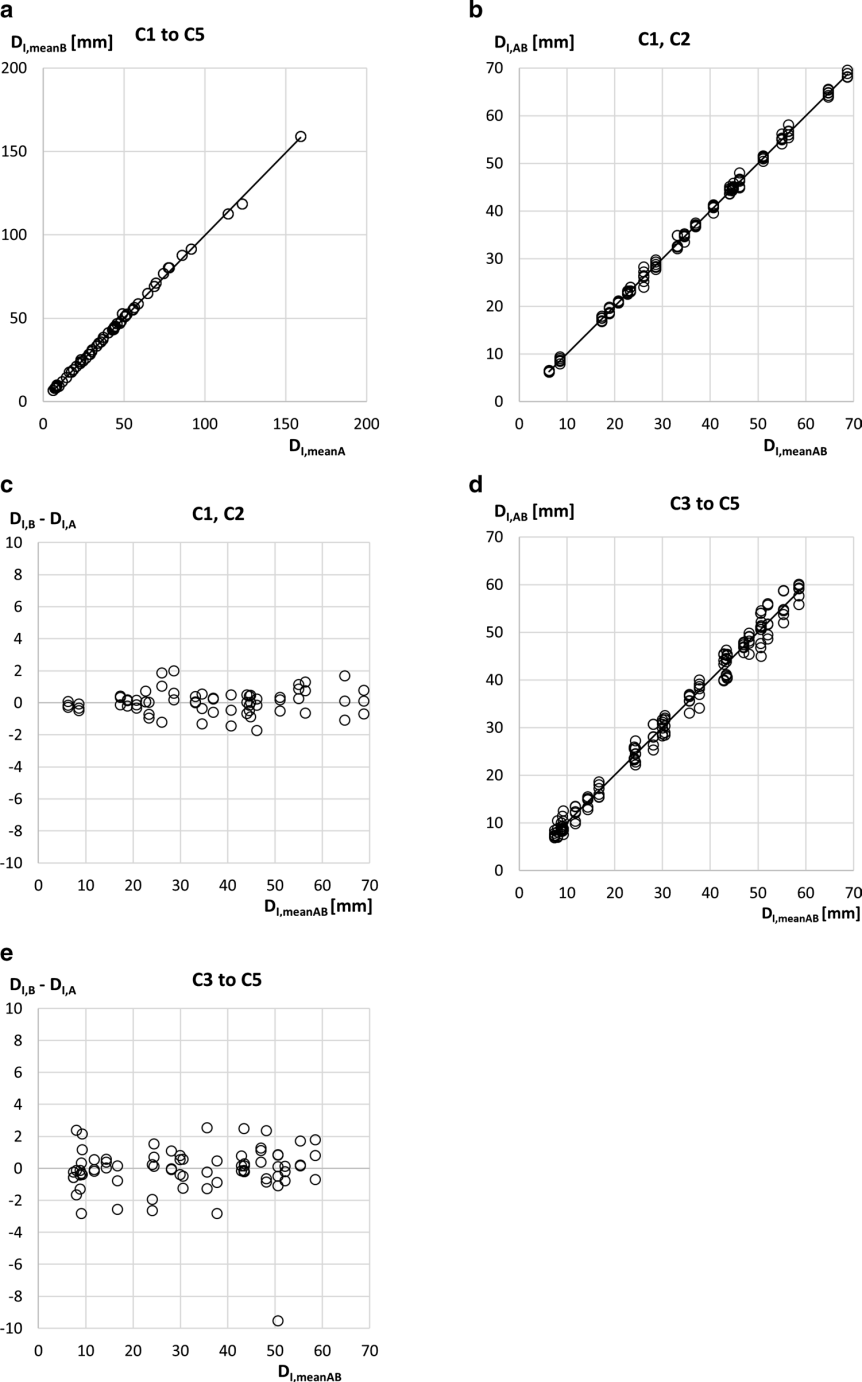
Intra-measurer reliability comparisons. **a** A sub-group of 56 athletes was measured twice. Measurement series A and B of the three measurers are compared (Note: not to be mixed up with the “A” used in the McGraw and Wong convention for calculating the ICC values). Results of series B (mean D_I_ values of the three measurers) are compared to series A. Each of the series A and B scores is based on three measurements at each of the eight sites in 56 athletes (this amounts to 2688 measurements of SAT thicknesses d_I_). ICC(A,k) = 0.999, SEE = 1.2 mm, limit of agreement (LOA) = 2.3 mm, and *ρ *= 0.999. **b** Experienced examiners only (C1–C2): a sub-group of 22 athletes from centres C1 and C2 and D_I_ below 70 mm. ICC(A1)_M1_ = 0.999, ICC(A1)_M2_ = 0.999, ICC(A1)_M3_ = 0.999, ICC(A1)_M1–3_ = 0.999 SEE = 0.7 mm, and *ρ *= 0.997. **c** Experienced examiners only (C1–C2): deviations D_I,B_–D_I,A_ of series A and B between the D_I_ measurements of the three measurers. SD = 0.7 mm; LOA = 1.4 mm. **d** Novice examiners only (C3–C5). A sub-group of 25 athletes from centres C3, C4, and C5 with D_I_ below 70 mm. ICC(A1)_M1_ = 0.997, ICC(A1)_M2_ = 0.997, ICC(A1)_M3_ = 0.991, ICC(A1)_M1-3_ = 0.995, SEE = 1.8 mm, and *ρ *= 0.988. **e** Novice examiners only (C3–C5). Deviations D_I,B_–D_I,A_ of series A and B between the D_I_ measurements of the three measurers. SD = 1.6 mm; LOA = 3.1 mm

All individuals (*N *= 56) participating in the intra-measurer reliability study (measurement series B compared to the corresponding part of series A) are included in Fig. 5a. The mean scores for the three measurers obtained in series B are compared to the means of series A: *R*^2^ = 0.999, SEE = 1.2 mm, LOA = 2.3 mm, *ρ *= 0.999, and ICC(A,k) = 0.999.

The results shown in Fig. 5b–e were obtained for athletes with SAT thickness sums D_I_ below 70 mm. The results shown in Fig. 5b, c were obtained in the sub-group of 22 athletes from centres C1–C2 (each athlete measured twice by the three measurers; median BMI = 22.4, range 19.2–27.9, IQR = 1.4 kg m^−2^), while the results shown in Fig. 5d, e were obtained for the sub-group of 25 athletes from centres C3–C5 (median BMI = 22.0, range 18.0–26.5, IQR = 3.3 kg m^−2^).

Figure 5b shows the measurement results of the three experienced measurers (from C1 to C2) in both series A and B (i.e., six individual measurements of the D_I_ values in each participant): *R*^2^ = 0.999, SEE = 0.7 mm, *ρ *= 0.997, and ICC(A,1)_M1_ = 0.999, ICC(A,1)_M2_ = 0.999, ICC(A,1)_M3_ = 0.999, and ICC(A,1)_M1–3_ = 0.999. Figure 5c shows the differences in D_I_ between the two measurement series A and B for each of the three experienced measurers plotted against the mean scores: SD = 0.7 mm, and LOA = 1.4 mm. In addition (not shown in the figure), D_E_ scores were also compared: SD = 1.1 mm and LOA = 2.2 mm.

Figure 5d shows the results of the three novice measurers from each of the centres C3–C5 in both series A and B (i.e., six individual measurements of the D_I_ values in each participant): *R*^2^ = 0.989, SEE = 1.8 mm, *ρ *= 0.988, ICC(A,1)_M1_ = 0.997, ICC(A,1)_M2_ = 0.997, ICC(A,1)_M3_ = 0.991, and ICC(A,1)_M1–3_ = 0.995. Figure 5e shows the differences in D_I_ between the two measurement series A and B for each of the three novice measurers plotted against the mean scores: SD = 1.6 mm, and the LOA = 3.1 mm. D_E_ scores: SD = 1.4 mm, LOA = 2.8 mm.

We observed a significant difference in the measurement deviations between experienced and novice measurers for the absolute values of differences D_I,B_–D_I,A_ (*p *< 0.05) and also for the absolute values of differences D_E,B_–D_E,A_ (*p *< 0.01).

**Fig. 6:**
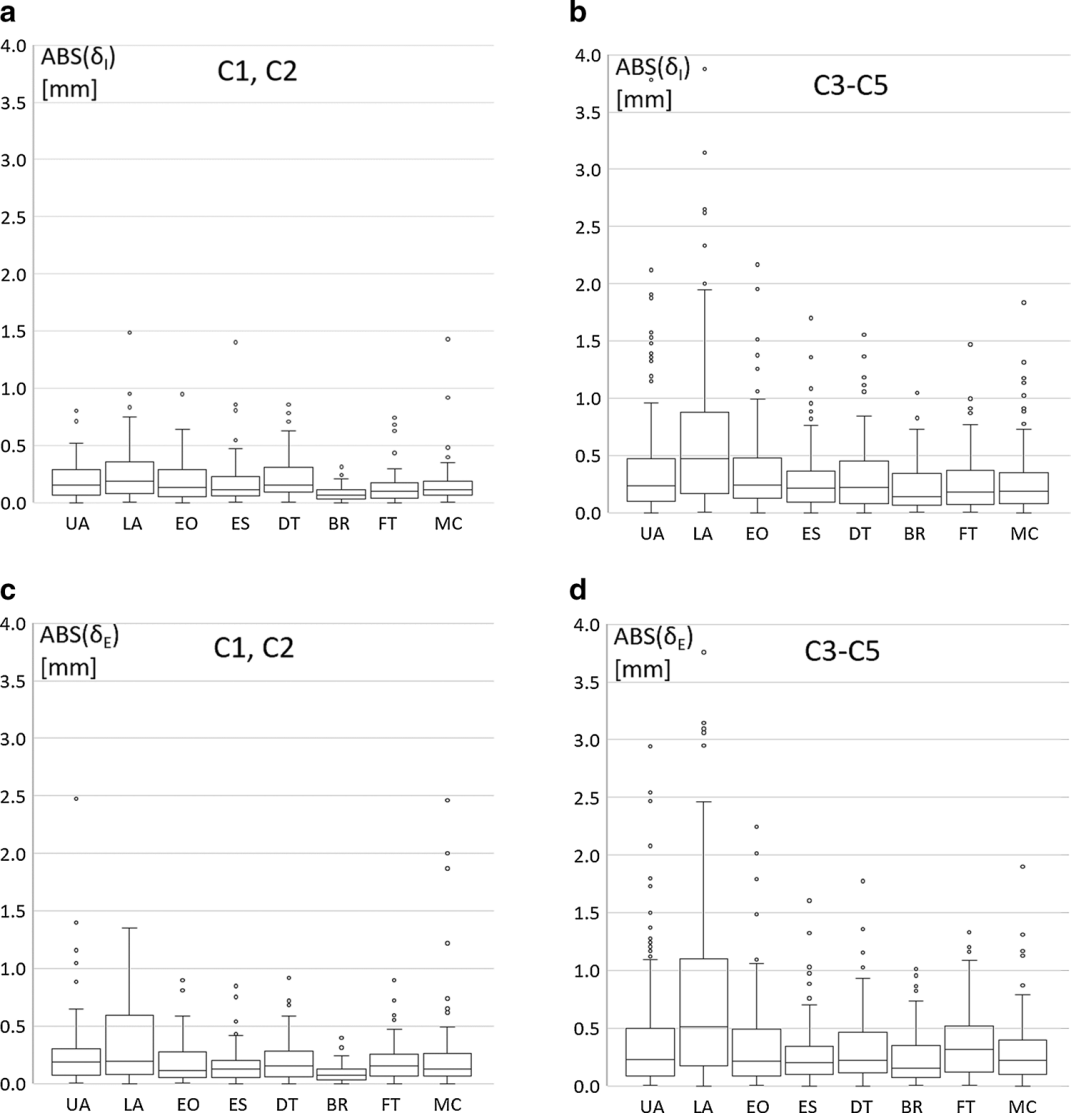
Absolute values of the measurement differences ABS(*δ*) of the three measurers from their mean at the individual eight sites. Data are presented in the Electronic Supplementary Material (Tables A3 and A4). *UA* upper abdomen, *LA* lower abdomen, *EO* external oblique, *DT* distal triceps, *BR* brachioradialis, *LT* lateral thigh, *FT* front thigh, *MC* medial calf. Index “I”: fibrous structures included, index “E”: fibrous structures excluded. **a** Experienced examiners (C1, C2): ABS (*δ*_I_) for each of the eight sites. The number of comparisons at each of the eight sites is: *N *= (16 + 16)3 = 96. **b** Novices (C3–5): ABS (*δ*_I_) for each of the eight sites. The number of comparisons at each of the eight sites is: *N *= (12 + 16 + 16)3 = 132. **c** Experienced examiners: ABS (*δ*_E_) for each of the eight sites. *N *= 96. **d** Novices: ABS (*δ*_E_) for each of the eight sites. *N *= 132

For studies of fat patterning in athletes, it is of relevance to analyse the measurement differences of the three measurers at the individual sites. These differences *δ* (absolute values) of the three measurers from their mean are shown in the box plots of Fig. 6a–d. Measurement differences were smaller at all sites in the expert group compared to the novices for both ABS(*δ*_I_) and ABS(*δ*_E_). The according box plot data (Tables A3 and A4) and the measurement differences in terms of percentages of the SAT thicknesses are presented in the ESM (Tables A5 and A6; Figs. A1a–d).

## Discussion

The median thickness measurement deviations at the individual eight sites (inter-measurer reliability study) were all below 0.2 mm when experienced measurers performed the measurements (with sufficiently high probe frequency of about 12–18 MHz). This is comparable to the (physically given) accuracy of ultrasound distance measurement, which is mainly determined by the wavelength-dependent image resolution, provided that the correct speed of sound for the pulse-echo thickness calculation in a given tissue is used. Therefore, sums of subcutaneous adipose tissue (SAT) thicknesses can be determined with high accuracy and reliability: the 95% limit of agreement for the experienced measurers was below D_I_ = 1.5 mm (and below D_E_ = 2.2 mm). This enables monitoring changes of SAT mass in athletes (which forms the dominant part of total body fat) with an accuracy of about 0.2 kg.

In female elite athletes, median SAT thickness sums D_I_ were three times higher as in their male counterparts (51 mm vs 17 mm). Before, only preliminary data comparing men and women have been presented [25]. B-mode ultrasound is the only imaging technique that enables also to quantify the amount of fibrous structures embedded in the SAT (fasciae). In this research, the embedded fasciae were quantified for the first time in a large group (*N *= 76) of elite athletes of various sports. The amount of these connective tissues was significantly lower in the 39 female elite athletes (median: 11%) when compared to the 37 male elite athletes (18%); this further increases the ratio of subcutaneous fat in elite female athletes with respect to that in male athletes. This has not been studied before, and also comparisons of SAT amounts in male and female elite athletes have been missing; only preliminary data of a small group of non-elite athletes were available [3] and exemplary comparisons of four elite athletes [6].

For persons with the same sitting height (i.e., similar leg length), the BMI and the MI, which is a sitting height corrected BMI, are identical (definition of the MI [15–17]).

Means of BMI and MI in large groups, which represent mean sitting height can, therefore, be expected to be similar. Median BMI and MI were 22.6 kg m^−2^ and 22.2 kg m^−2^, respectively. The small difference in our group may be because a part of the measurements were made in a Hispanic country, where sitting height medians are higher when compared to Caucasian White persons [26]; this results in MI values lower than BMI values. However, the difference between BMI and MI was large in several cases (up to 1.7 kg m^−2^); a body mass change of more than 5 kg would correspond to a BMI difference of 1.7 kg m^−2^. Such differences are of core relevance for both assessing the athlete’s health status and for designing competition rules based on ‘relative body mass’ (such rules are currently used in ski jumping, for example [15, 17], where the BMI is used).

### Body Fat Measurements in Sport

The status of body composition assessment in sport has been reviewed by the Working Group on Body Composition, Health and Performance (under the auspices of the IOC Medical and Scientific Commission) [2], and best practice protocols for physique assessment in sport were recently presented, including the standardised US method, which is capable of measuring SAT at an accuracy level not reached by any other method [27]. All other methods analysed there are usually not sufficiently accurate for monitoring body composition on the fine scale needed in top-level athletes. This is particularly the case if athletes are excessively small, large, or lean [2], because most athlete groups are highly specialised and their sport-specific physique imperatives are not in line with general morphological norms [27–31]. Therefore, many of the assumptions upon which measurement techniques are based are not valid in athletes. Densitometry, for example, has resulted in scores of minus 12% fat [28], and with DXA, the seven leanest in a group of male athletes showed negative fat on the torso [29]. Obviously, the morphology assumed in the measurement algorithms causes impossible results in lean athletes. Limitations of measurement techniques are discussed in the ESM and in the literature [2, 3, 6, 30–32].

### Ultrasound Brightness-Mode Imaging and Distance Measurement Accuracy

Diagnostic (brightness-mode) ultrasound has been used for fat measurement since 1965 [33, 34], and many publications followed. At sufficiently high probe frequency (12–18 MHz), the thickness measurement accuracy is approximately 0.1–0.2 mm [3, 6, 35], provided that the appropriate speed of sound in the given tissue is used (1450 ms^−1^ in fat [18–22]). The high accuracy enables measuring the embedded fibrous structures, which amount to substantial percentages of the SAT (Fig. 2c, d, Tabl.1, and ESM: Tables A1 and A2). A typical US image of SAT at the ‘front thigh’ site is shown in Fig. 1b. A thick layer of gel between the probe and the skin (black band above the epidermis in Fig. 1b, c) avoids compression. This is an important feature of this US measurement technique [3–7] as adipose tissue is highly compressible, and this degree of compressibility varies from site to site and between individuals [3]. Factors influencing accuracy are analysed in the ESM and in various publications [3, 6, 22]. However, the technical accuracy limits for US are not the crucial point: the limitations are set by biological reasons including detection of furrowed borders and visco-elastic deformations of adipose tissue. Therefore, measurement reliability is the overall limiting factor (Table 1).

**Table 1:** Data of athlete groups

Groups	*m* (kg)	*h* (m)	*s* (m)	*l* (m)	MI (kg m^−2^)	BMI (kg m^−2^)	D_I_ (mm)	D_E_ (mm)	D_F_ (mm)	D_F, %_ (%)	*N*
ALL											
Median	68.2	1.731	0.931	0.953	22.2	22.6	35.1	30.4	4.0	13.3	76
IQR	14.3	0.126	0.060	0.089	2.4	2.8	35.8	32.8	3.4	8.5	
Q1	60.3	1.677	0.899	0.920	21.2	21.1	17.1	13.7	2.3	9.9	
Q3	74.6	1.802	0.959	1.009	23.6	24.0	52.9	46.5	5.8	18.4	
ALL_m											
Median	72.0	1.799	0.946	0.992	23.2	23.1	17.2	13.7	2.4	18.3	37
IQR	14.5	0.098	0.051	0.069	1.9	2.1	26.7	24.2	3.2	8.1	
Q1	66.3	1.742	0.919	0.960	22.1	22.0	8.8	6.9	2.1	13.6	
Q3	80.8	1.840	0.970	1.029	24.0	24.1	35.5	31.1	5.2	21.7	
ALL_f											
Median	61.3	1.690	0.903	0.928	21.6	22.1	51.1	44.0	4.8	10.5	39
IQR	13.8	0.068	0.056	0.069	2.1	3.4	41.7	40.7	3.0	5.8	
Q1	56.2	1.660	0.883	0.881	20.5	20.3	31.9	27.9	3.4	6.8	
Q3	69.9	1.728	0.939	0.950	22.5	23.7	73.6	68.6	6.4	12.7	
ALL_w											
Median	62.8	1.705	0.900	0.941	21.9	21.9	24.4	19.2	3.3	16.0	36
IQR	13.9	0.128	0.069	0.075	2.0	2.2	28.9	25.8	3.4	6.8	
Q1	57.0	1.662	0.872	0.913	21.2	20.8	9.0	7.0	1.9	12.8	
Q3	71.0	1.790	0.940	0.989	23.2	22.9	37.9	32.7	5.3	19.7	
ALL_nw											
Median	71.1	1.741	0.942	0.966	22.8	23.4	50.3	44.8	4.4	10.1	40
IQR	12.3	0.121	0.051	0.084	2.9	2.5	46.7	43.7	3.1	6.7	
Q1	64.9	1.698	0.917	0.928	21.1	22.0	25.5	22.9	3.0	6.9	
Q3	77.2	1.819	0.968	1.013	24.0	24.5	72.1	66.6	6.1	13.5	

Groups: *ALL* all 76 athletes, *m* males, *f* females, *w* weight-sensitive sports, and *nw* non-weight-sensitive athletes. Measured anthropometric data: *m* body mass, *h* stature, *s* sitting height, *l* leg length. Anthropometric indices: *MI* mass index, and *BMI* body mass index, *D* sum of subcutaneous adipose tissue thickness; further abbreviations: *I* fibrous structures included, *E* excluded, *F* fibrous structures, *F,%* percentage of fibrous structures with respect to d_I_ (thickness of the SAT at the given site), *N* number of athletes, *Q* quartile, D_I_, D_E_, D_F_, and D_F,%_ were calculated from the mean of the three measurers’ values

Data of all individuals are presented in Table A1 (Electronic Supplementary Material)

### Reliability of the Standardised US Method: An Overview

US images can never be captured by different investigators or at different times by the same investigator at exactly the same US probe position and orientation, which affects reliability. Therefore, a standardised technique has been introduced recently [6, 7]. More information about the choice of standard US sites can be found in the ESM, and in previous publications [3, 4, 6, 7]. Reliability obtained by experienced measurers has been tested in groups ranging from lean [4, 6] to overweight and obese [7]; reliability has also been tested in children [10, 13]. However, the extent to which measurer experience plays a role has never before been analysed systematically.

Tables 2 and 3 compare the core results obtained previously by experienced measurers [4, 6, 7] to the findings of the current multicentre study (MCS), in which both experienced and novice measurers were involved in the inter- and intra-measurer reliability studies. Experienced measurers of centres C1–C2 had their US system permanently available, whereas the novices (C3–C5) had to borrow a US system for their measurement series and they had no preceding experience with US imaging. Their training was limited to a 2-day course, followed by supervised US measurements in about five individuals. These are main factors causing the lower accuracy and reliability obtained by the novices.

**Table 2:** Inter-measurer reliability

Study reference	D_I_–range	95% LOA D_I_	95% LOA D_E_	Median of ABS (∆D_I_)	Median of ABS (∆D_E_)
2016 [6]	10–51	± 1.1	± 1.3	0.24	0.36
MCS experienced	6–70	± 1.2	± 1.7	0.30	0.38
MCS novices	6–70	± 3.1	± 4.0	0.97	1.26

Comparison of previous results [6] to the results obtained by the experienced (C1–C2) and the novice measurers (C3–C5) of this multicentre study (MCS). Results obtained in the sub-group consisting of 37 male and 28 female participants (30 of C1–C2, and 36 of C3–C5) with D_I_ < 70 mm are shown. *D* sum of SAT thicknesses (fibrous structures included: index I; excluded: index E), *LOA* limit of agreement [23]. All values in mm

**Table 3:** Intra-measurer reliability

Study reference	D_I_–range	95% LOA D_I_	95% LOA D_E_	Median of ABS (∆D_I_)	Median of ABS (∆D_E_)
2017 [7]	12–77	1.4	1.6	0.43	0.41
2017 [7]	44–245	2.9	3.8	0.89	0.89
2017 [7]	12–245	2.2	3.2	0.61	0.59
MCS experienced	6–70	1.4	2.2	0.39	0.57
MCS novices	6–70	3.1	2.8	0.55	0.89

Comparison of previous results [7] to the results obtained by the experienced (C1–C2) and the novice measurers (C3–C5) of this multicentre study (MCS). Results obtained in the sub-group consisting of 25 male and 22 female participants (22 of C1–C2, and 25 of C3–C5) with D_I_ < 70 mm are shown. *D* sum of SAT thicknesses (fibrous structures included: index I; excluded: index E), *LOA* limit of agreement

All values in mm

Measurement deviations of experienced measurers in the current study (95% LOA was ± 1.2 mm for D_I_ that ranged from 6 to 70 mm) did not differ noticeably from previous results (± 1.0 mm, at D_I_ ranging from 10 to 51 mm, [6]). However, the deviations of the novice measurers were substantially larger, indicating clearly that measurers need sufficient experience to obtain the highest accuracy and reliability-level possible. The reasons for the larger errors were: bad US image quality, the US probe was not exactly at the marked position, incorrect interpretation of embedded structures as being muscle fasciae (e.g., Camper’s fascia [3, 4]), the ROI not set symmetrically, or the gel layer not thick enough resulting in fat compression. Another source of error may be that some participants did not stop breathing at mid-tidal expiration when US images were captured.

The inter- and intra-measurer deviations were larger when thicker SAT layers were measured; however, the relative deviations (ΔD_I_/D_I_) were found to be smaller with increasing SAT thicknesses [7]. In most cases, the deviations with respect to D_E_ (fibrous structures excluded) are slightly larger, because for measuring D_E_, several tissue borders within the SAT need to be detected additionally. In the inter-measurer reliability tests, the deviations for novice measurers were about three times larger than for the experienced measurers, but in the intra-measurer reliability tests, this difference was only twofold, indicating that novices repeated some of their measurement mistakes.

### Reliability at Individual Measurement Sites

The reliability of the sum D of the eight SAT thicknesses d is composed of the reliabilities of the thickness measurements at the individual sites. Figure 6a–d shows the absolute values ABS(*δ*) of the measurer differences from their means at the eight sites (ESM: Tables A3 and A4). Median values, interquartile ranges (IQR), and third interquartile values (Q3) were substantially smaller in the group C1–C2 (experienced examiners) compared to C3–C5 (novices) at all sites. At sites with usually higher SAT thickness d, differences ABS(*δ*) also tended to be higher, but all medians of the experienced group were below 0.2 mm, and below 0.5 mm in the novices’ group. Not only the differences ABS(*δ*), in mm, but also the relative differences ${\text{ABS}}\left( {\delta_{\text{rel}} } \right) = 100\, \cdot \,{\text{ABS(}}\delta )/\tt\text{d}$, in % of the SAT thickness d at the given site, are of relevance. For example, ABS(*δ*) is low for EO, but the according ABS(*δ*_rel_) has the highest value of all sites (ESM: Tables A5 and A6). This is one of the reasons why this site has meanwhile been replaced by lateral thigh (LT) [6]. Another reason is that the site EO causes measurement problems in obese individuals [7].

A further reason for replacing the site EO by LT is that the latter is a pronounced fat depot site in women and thus of high relevance when studying sex differences. The measurement deviations at the site LT (median of absolute deviations was 0.24 mm, median SAT thickness was 14 mm; corresponding to 1.7%) found in an intra-measurer reliability study published in 2017 [7] were comparable to the measurement deviations which these authors found at UA and LA (0.21 mm and 0.26 mm, 12 mm and 19.5 mm; 1.8% and 1.3%, respectively). The participants studied in the cited publication [7] ranged from extremely lean to obesity class III. Based on these findings, the measurement differences at LT in our study group can be assumed to be in a similar range as found at the abdomen sites.

### SAT Thickness Measurement Errors Transform Linearly into Fat Mass Errors

The small error of US thickness measurements of a fat layer transforms linearly into the error of subcutaneous fat mass, because the fat volume is proportional to the (calibrated) mean of subcutaneous fat thickness of the whole-body surface. An SAT thickness measurement error of 1.4 mm (95% LOA; see Tables 2 and 3, and Figs. 3, 4, 5) transforms into an SAT mass error of about 0.2 kg (see ESM); this is almost an order of magnitude below the daily body weight fluctuations. SAT makes by far the largest part of total body fat (typically 80–90% of anatomically detectable fat mass [36]). The SAT thickness sums in females can be expected to be higher when the site EO is replaced by LT [7, 25, 37].

None of the measurement techniques for cross-sectional or longitudinal studies of body fat is capable of measuring on such a fine scale as US [2, 27], and no other can quantify the amount of connective tissues embedded in the SAT (‘fascias’), which forms a substantial part of SAT (4.0 to 29.3% in the group of elite athletes studied here).

### Relative Body Mass: BMI and MI

Several indices that are power functions of body mass (m) and stature (h) were originally meant for measuring body fatness [38–40]. One such index that is widely used is the body mass index (BMI or Quetelet’s index): BMI = *m/h*^*2*^. Figure 2a shows that the BMI is useless for assessing body fat in athletes: as expected [2], there was no correlation between BMI and SAT thicknesses sums. Similar results were found in several other groups, too [7, 11, 37]. Conversely, among anorexia nervosa patients, with extremely low BMI (below 17.5 kg m^−2^), some individuals may have subcutaneous fat thickness values comparable to those of healthy women [9, 12]. When using the BMI as a measure of ‘relative body mass’, there is a further important limitation that the World Health Organisation (WHO) Expert Committee on Physical Status has pointed out:

“Problems arise, however, in adults whose shape differs from the norm… Care should therefore be taken in groups and individuals with unusual leg length to avoid classifying them inappropriately as thin or overweight” [14]. Based on this justified critique, a measure for relative body mass, the mass index MI has been developed [15, 17]: ${\text{MI}}_{1} = 0.53\;{{m}}/({{hs}})$. This measure considers not only stature *h*, but also the individual’s sitting height *s* (and thus, implicitly, the leg length *l*). For the derivation of the MI_1_ formula, see ESM. In this study, mean BMI was 22.6 kg m^−2^ and mean MI was 22.2 kg m^−2^, the difference MI_1_-BMI was large in several individual cases, ranging from − 1.7 to 1.3 kg m^−2^. Particularly in weight-sensitive sports, such differences in individuals are of core relevance for assessing the athlete’s health status and for rising the alarm when the individual’s body weight becomes critical [1].

### Characteristics of the Athlete Groups and Their SAT

Figure 2a shows that there was no correlation (*R*^2^ = 0.13) between BMI (which ranged from 17.9 to 29.0 kg m^−2^) and SAT thicknesses D_I_ (ranging from 6 to 160 mm). This also holds true for the MI_1_ (*R*^2^ = 0.09). Neither BMI nor MI_1_ give useful information about athletes’ body fat. Although relative body mass was 1.0 kg m^−2^ lower in females in terms of BMI (and 1.6 kg m^−2^ in terms of MI), their median D_I_ was 3.0-times higher (51.1/17.2 = 3.0). In addition, their median percentage of embedded fibrous structures was 1.7 times lower than in males: therefore, females’ median D_E_ was 3.2 times the value found in males (Fig. 2c, d; Table 1). In the sub-group of athletes in weight-sensitive sports, women (median D_I_ = 33.1 mm) had about 3.5 times the amount of SAT as men (median D_I_ = 9.5 mm), and for athletes in the non-weight-sensitive group, females’ median D_I_ (66.7 mm) was 2.9 times higher than that in males (D_I_ = 23.1). Using LT instead of EO would further increase the ratio because LT is a prominent fat depot site in women [25]. Four (of 39) D_I_ values of women were below 25 mm, and 15 (of 37) values of men were below 12 mm (“extremely low” according to [12]).

The means of all female participants were significantly higher for D_I_, D_E_, and D_F_, and significantly lower for D_F,%_ when compared to means of all male participants (*p* ≤ 0.001). The percentage of embedded fibrous structures tended to decrease with increasing D_I_ in both male and female participants (*R*^2^ = 0.35 and 0.41, respectively). The median percentage of fibrous structures for all athletes was 13.3% (4.0–29.3%), for male athletes 18.3% (8.9–29.3%), and for female athletes 10.5% (4.0–22.5%).

The difference in SAT between highly trained male and female athletes is large in most cases. This also holds true for total body fat (TBF), because SAT mass represents the major part of TBF (typically 80–90%) [36].

### Limitations

1. Visceral adipose tissue, which is typically about 10–20% of total body fat [36] (but may also be beyond this percentage range in some individuals), is not included in the US SAT measurement. This has to be considered when using SAT as a surrogate for total body fat.
2. Currently, only preliminary normative data are available for comparisons [12].

## Conclusion

Regarding the reliability of this US method, when the standardised brightness-mode US technique for measuring SAT is applied by experienced measurers in athletes (with low or moderate body fat: D_I_ < 70 mm), the 95% LOA can be expected to be below 1.5 mm for the sum of thicknesses from eight sites D_I_ (fibrous structures included), and below 2.2 mm for D_E_ (fibrous structures excluded). At the individual eight sites, median measurement differences (from their means) ranged from 0.06 to 0.19 mm (third quartiles: 0.11 to 0.36 mm).

The inter-measurer results found here in a large group of athletes of various sports are in line (Table 2) with a preliminary study [6] that compared the results of three experienced measurers obtained in a small group of lean athletes (*N *= 12). The standardised US method enables tracking of SAT thickness changes that correspond to about 0.2 kg changes in SAT mass, which is substantially below the daily body mass changes. Measurement differences of novice measurers were approximately three times larger. Their results are still useful; however additional training, particularly in US image capturing, is necessary to attain the highest possible level of reliability.

In terms of body composition, the US measurement results obtained in this group of elite athletes from various sports showed that the median SAT thickness sum D_I_ was three times higher in the elite female athletes as in the male group (51 mm vs 17 mm). In addition, the percentage of connective tissue embedded in the SAT was significantly (*p *< 0.01) lower in women (median 11%) than in men (18%) and percentages tended to decrease with increasing D in both groups. This also holds true when comparing female (12%) and male (15%) participants within the same D_I_ interval ranging from the lowest female to the highest male value (14–60 mm), although the D_I_, D_E_, and D_F_ values did not differ significantly in these sub-groups (*p *> 0.1). The standardised US method is the only measurement technique that has sufficient accuracy to quantify the amount of fibrous structures embedded in the SAT.

Comparing BMI to D_I_, there were no correlations between BMIs or MIs and SAT thickness sums D_I_ for this group of all 76 athletes from the five research centres (D_I_ ranged from 6 to 160 mm, and BMI from 18 to 29 kg m^−2^). The BMI is a measure of relative body mass, but not a useful tool to determine body fat. This also holds true for the MI, but this improved index of body mass considers the individual’s leg length. Differences (MI-BMI) were large in several cases and ranged from − 1.7 to + 1.3 kg m^−2^ (median BMI was 22.6 kg m^−2^), which supports the inclusion of sitting height (or leg length) in all basic data sets.

Comparing D_I_ to D_E_, the values of SAT sums with fibrous structures included (D_I_) are closely correlated with D_E_ (*N *= 76 athletes, for means of the three measurers’ values: *R*^2^ = 0.997, SEE = 1.5 mm); the slope of the regression line was lower in the male (0.88) than in the female group (0.96), indicating the higher percentage of embedded fibrous structures in male athletes.

*Future research* We encourage the application of this standardised US method for the study of body composition in athletes of various sports, and the use of these data sets for performance optimisation or medical diagnoses for sports in which low weight and body composition problems exist. Only preliminary data sets are currently available, so the question of what minimum fat level is acceptable from a medical point of view for an individual with unique genetics and lifestyle cannot be answered at this time. In a recent study of anorectic patients [9], the US method has shown that SAT amounts differed by 330%, although their (extremely low) BMI differed by only 12% when the 18 female patients were divided into two groups based on the group median of D_I_.

The standardised US method enables accurate studies of fat patterning. In this study, we primarily discussed the comparison of sums of SAT thicknesses from the eight sites between male and female athletes, but there is much more information contained in the distribution of fat in men and women of different sport groups, and in patients with chronic conditions. Other lines of research include intervention studies that effect body composition (e.g., studies on physical training effects or sports nutrition). Furthermore, it will be interesting to see how the US data compare to other established methods for measuring body composition, like the four-component model, MRI, or DXA. Such studies are in progress in some of the centres that participated in this study.

## Electronic supplementary material

Below is the link to the electronic supplementary material.

Supplementary material 1 (PDF 1286 kb)

## Abbreviations

BMI: Body mass index: BMI = *m*/*h*^2^ (kg m^−2^)
C: Cormic index
CT: Computer tomography (X-ray based)
$\tt \bar{C}$: Cormic index, mean
C1–C5: Research centres
d: SAT thickness at a given site (this is the average of the distances measured within the region of interest) (mm)
D: Sum of SAT thicknesses at all eight sites in a given participant (mm)
*δ*: Measurement deviation of each measurement (M1, M2, and M3) from the mean of three measurements at a given site in a given subject (mm)
*δ*_rel_: *δ*_rel_ = 100·*δ*/d_MEAN_ (%)
Δ: Deviation of the sum of eight sites from the mean of the sums of the three measurements in a given participant (mm)
E: Excluded; indicates that the fibrous structures are not included
ESM: Electronic supplementary material
F: Fibrous structures
F, %: Fibrous structures (%)
*h*: Stature (m)
I: Included; indicates that the fibrous structures are included
ISAK: International Society for the Advancement of Kinanthropometry
f: Female
*l*: Leg length, measured from the floor to the anterior superior iliac spine
m: Male; (also used as unit for length: metre)
*m*: Body mass (kg)
*m*_SAT_: Subcutaneous adipose tissue mass (kg)
MI: Mass index: ${\text{MI}}_{1} = 0.53\;{{m}}/({{hs}})$ (kg m^−2^)
MRI: Magnetic resonance imaging
nw: Non-weight-sensitive
*ρ*_fat_: Density of body fat
*s*: Sitting height (m)
SAT: Subcutaneous adipose tissue
US: Ultrasound
w: Weight sensitive

## Statistics

ABS: Absolute value of a number
MEAN: Mean value
*N*: Number of values
*R*^2^: Coefficient of correlation
ROI: Region of interest
*ρ*: Spearman’s rank-order correlation coefficient (Spearman’s rho)
SD: Standard deviation
SEE: Standard error of estimate

## US sites (order: trunk, arms, legs)

UA: Upper abdomen
LA: Lower abdomen
EO: External oblique
ES: Erector spinae
DT: Distal triceps
BR: Brachioradialis
FT: Front thigh
MC: Medial calf
LT: Lateral thigh
