# Supplementary material for: Relative Body Weight and Standardised Brightness-Mode Ultrasound Measurement of Subcutaneous Fat in Athletes: An International Multicentre Reliability Study, Under the Auspices of the IOC Medical Commission
Source: Sports Med. 2019 Sep 30;50(3):597–614. doi: 10.1007/s40279-019-01192-9 (PMC7018793; doi:10.1007/s40279-019-01192-9)
Supplement: Supplementary file 1 — Supplementary material 1 (PDF 1286 kb) [file 40279_2019_1192_MOESM1_ESM.pdf]

Electronic Supplementary Material
(ESM)

Relative body weight and standardised ultrasound measurement of subcutaneous fat in athletes: an international multicentre reliability study, under the auspices of the IOC Medical Commission
(Sports Medicine)

Wolfram Müller, Alfred Fürhapter-Rieger, Helmut Ahammer, Timothy G. Lohman, Nanna L. Meyer, Luis B. Sardinha, Arthur D. Stewart, , Ronald J. Maughan, Jorunn Sundgot-Borgen, Tom Müller, Margaret Harris, Nuwanee Kirihennedige, Joao P. Magalhaes, Xavier Melo, Wolfram Pirstinger, Alba Reguant-Closa, Vanessa Risoul-Salas, Timothy R. Ackland

Corresponding author:
Wolfram Müller
Medical University of Graz, Biophysics
Neue Stiftingtalstraße 6
8010 Graz, Austria
Email: wolfram.mueller@medunigraz.at
Phone: ++43 316 380 3913

Electronic Supplementary Material

Ad: Obtainable accuracy of US thickness measurements

The accuracy obtainable with brightness-mode (B-mode) US depends on the probe frequency (high frequency increases resolution, but reduces image depth due to increased attenuation), on the appropriate setting of the US system (primarily: gain, time-gain compensation, image depth, number and position of foci, image analysis parameters), and on the skills of the investigator (small movements or tilts of the probe can change image quality substantially). Linear probes should be used for quantitative measurements. Tissue compression can be avoided by including a thick layer of gel between the probe and the skin [3,6]. The resolution of US imaging is determined by the US wavelength (λ): at 18 MHz probe frequency (f), which is the best choice for the thin subcutaneous adipose tissue layers found in most athletes, a resolution approximately equal to the wavelength (0.1 mm) can be obtained because diffraction and spatial length of the US pulse are the limiting factors for transverse and longitudinal resolution: $\lambda = c/f = 1450\ \mathrm{m\,s^{-1}}/1.8 \cdot 10^7\mathrm{s^{-1}} \approx\ 0.1\ \mathrm{mm}$. A sound speed deviation from the real speed in the given tissue of 30 $\mathrm{ms^{-1}}$ would result in 2% thickness measurement error. For example, a 10 mm thick layer would erroneously be measured as 10.2 mm. Sound velocity data can be found in [18-21] and detailed discussions of the US thickness measurement accuracy can be found in [3,6,22].

Note: It is not the technically given high accuracy that limits the usefulness of the US method, but rather biological reasons (furrowed borders and visco-elastic deformations of adipose tissue) mostly affect the reliability. The technically given accuracy of about 0.1 mm cannot be outperformed by any other method (including mechanical measurements of tissue thickness layers using a micrometre screw [2]).

Choice of US sites

Deviations of repeated measurements depend primarily on the micro-anatomical structure at a given site (provided that the measurers are trained sufficiently). In most cases, these deviations are larger than the technically given accuracy limitations. To obtain the highest possible reliability, it is of paramount importance to choose US sites where SAT thickness is mostly constant around the centre of the site, to choose sites

that can be easily and reliably marked, and to capture the US images in standardised positions (because fat is visco-elastic) [3,4,6,7]. The sites selected for the standardised US measurement of SAT obey these criteria and they represent trunk (3 sites), arms (2 sites), and legs (3 sites).

Linear transformation of SAT thickness measurement error into fat mass error

The small errors resulting from the accuracy and reliability limitations of US thickness measurements of SAT layers transform linearly into the error of subcutaneous adipose tissue mass m_{SAT} because the fat volume is proportional to the (calibrated) mean subcutaneous fat thickness ($d_{mean,cal}$): $m_{SAT} = S\, d_{mean,cal}\, \rho_{fat}$, with S being the surface area, and ρ_{fat} the density of fat tissue. As usual with skinfolds [31], we believe that the sum of thicknesses should be used directly for comparisons between athletes because any calculation of total body fat mass (or of total SAT mass) involves model assumptions and includes possible additional measurement errors (surface area, mean SAT thickness calibration, density of SAT in various body regions and individuals, changing water content). However, for a rough estimate in an athlete with a body surface area of $A=2.0\ m^2$, a D_I-value of 8 mm would result in about 1.4 kg SAT. For this approximate calculation, a factor of 0.7 was used for calibrating the mean SAT thickness derived from the eight sites [22] (this considers that the standardised sites over-represent the mean SAT thickness because these sites were chosen to represent typical fat patterning, but not mean SAT thickness). From this approximate assessment of fat mass, we learn that a measurement error of 1.4 mm (95% LOA; see Tables 2 and 3) transforms into a SAT mass error of less than 0.2 kg.

Body fat measurements in sport:

In all computed tomography methods, the accuracy of volumetry (and thus the determination of fat mass) depends strongly on the choice of the image segmentation parameters and the measurement parameter settings. Additionally, MRI pixel size used for total body scans (typically 1.3-2.0 mm) is not small enough for measuring the thin fat layers in lean athletes with sufficient accuracy. X-ray computer-tomography (CT) cannot be used for routine measurements because of the high radiation exposure. Skinfolds (SF) measure a compressed double layer of SAT and skin: a comparison of highly accurate US thickness measurements of SAT to SF showed that compressibility

of SAT and also skin thickness (variation of 300%) largely depends on the anatomical site and on the individual [3,30]. This explains the accuracy limitations of SF methods, which can be severely misleading [3]. Fat mass calculations for individuals based on SF is outdated because of excessive errors [31]. Similarly, large errors result from the widely used bioimpedance (BIA) method [2,8,27], which is based on a highly reductionist measurement concept that aims to assess body fat mass by simply measuring the alternating current resistance (e.g., at 50 kHz) of the human body [2,27]. For detailed discussion of methods in use, see [2,8,27].

BMI and the derivation of the MI formula for relative body weight:

The body mass index (Quetelet's index) $\mathrm{BMI} = m/h^2$ (*m* body mass, *h* stature) is not useful for assessing body fat of individuals, particularly in athletes (compare to Fig. 2a in the main text). When using the BMI for assessing 'relative body mass', there is a further important limitation: the BMI ignores individual body properties [14]. Therefore, W. Müller has developed an improved measure for relative body weight, which considers the sitting height *s* (and thus, implicitly, also the leg length *l*): the mass index MI [15,16,17]. The MI is a modified BMI. The general formula for this modified BMI is:

$\mathrm{MI} = \mathrm{BMI}\ (\overline{\mathrm{C}}/\mathrm{C})^k$, or, explicitly: $\mathrm{MI} = \frac{m}{h^2}\left(\frac{\overline{\mathrm{C}}}{\frac{s}{h}}\right)^k$

The exponent *k* weights the modification term. The Cormic index $\mathrm{C} = s/h$. At a given *h*, a large *s* is associated with a low *l* (according to *h=s+gl; g* is a geometry factor of the individual). Therefore, both *s* or *l* can be used for designing a measure that considers the individual's geometric properties. $\overline{\mathrm{C}} = 0.53$ represents mean sitting height (this reference value is chosen in the middle of the Cormic index continuum). The choice of $k = 1$ (thus 'MI$_1$') takes both into consideration, the dependency of relative body weight on stature h and on sitting height s:

$\mathrm{MI}_1 = 0.53 \cdot \frac{m}{hs}$.

MI$_1$ is chosen symmetrically between $\mathrm{MI}_2 = 0.53^2 \cdot m/s^2$ ($k = 2$ would ignore the impact of stature h on relative body mass as h would cancel out in this case), and $k = 0$ ignores the impact of sitting height s (and thus of leg length, too): $\mathrm{MI}_0 = m/h^2 \equiv \mathrm{BMI}$. For a person with long legs, the MI is higher than the BMI, and vice versa for a person with short legs. With the same stature h, a person with shorter legs (large sitting height) can be expected to have higher body mass *m* because his volume

is higher due to the relatively large dimensions of his upper body. Particularly in low weight sports, assessing 'relative body mass' of athletes is a crucial health parameter. A BMI of 17.5 kgm^{-2} is one of the four criteria for diagnosing anorexia nervosa: when using the MI instead of the BMI, different diagnoses would result whether the MI is equal to the BMI (due to mean leg length), or differs by one or even more units. In the group of elite athletes described in this publication, differences MI-BMI ranged from -1.7 kgm^{-2} to +1.3 kgm^{-2}. At a BMI of 17.5 kgm^{-2}, a difference of -1.7 kgm^{-2} would result in 15.8 kgm^{-2}, which is far below the weight criterion for anorexia nervosa, whereas +1.3 kgm^{-2} would result in 18.8 kgm^{-2}, which is far above this criterion, and even above the WHO criterion for underweight (which is 18.5 kgm^{-2}). In addition to using the MI, the accurately measured SAT amount should be included in diagnostics and therapeutics of low weight problems in athletes and in anorexia nervosa patients [9].

The MI is defined such that the WHO cut-off points for underweight, overweight, and obesity (18.5, 25, and 30 kgm^{-2}) can remain the same when replacing the BMI by the MI: their means are equal for groups with a mean Cormic Index of C = 0.53. The most important advantage of the MI over the BMI is the appropriate assessment of relative weight of the individual, although group means may be similar or the same. In the group of mainly Caucasians (and a small number of Hispanics) studied here, median BMI was 22.6 kgm^{-2}, and median MI 22.2 kgm^{-2}. Using the MI_1 instead of the BMI will also contribute to the discussion about BMI cut-off points for ethnic groups with shorter or longer legs [26]. These populations show large differences between MI and BMI.

Table A1: Individual data of all 76 participating athletes.
The same abbreviations as in Table A2 are used here. Additionally: w (weight-sensitive), and nw (non-weight-sensitive). Ordered according to increasing body mass index (BMI).

Table A2: Data of athlete sub-groups
Groups: all 76 athletes (ALL), males (m), females (f), weight sensitive sports (w), and non-weight-sensitive athletes (nw). C1 to C5 indicate the athlete sub-groups of the five participating centres. Anthropometric data: body mass (*m*), stature (*h*), sitting height (*s*), leg length (*l*). *Anthropometric* indices: mass index MI, and body mass index BMI. D: sum of subcutaneous adipose tissue thickness. Further abbreviations: mean: mean of the values of the three measurers; I: fibrous structures (i.e., fasciae) included; E: excluded; F: fibrous structures (fasciae); F,%: percentage of fibrous structures (fasciae) with respect to D_I. N is the number of athletes. Note: not all data sets are normally distributed. For median values of the main groups, see Table 1 (in the main text). Data are sorted according to increasing BMI.

Table A1

m	*h*	*s*	*l*	MI	BMI	MI-BMI	$D_{I,mean}$	$D_{E,mean}$	$D_{F,mean}$	$D_{F,\%}$	Age	Sex		Sport		Sports
[kg]	[m]	[m]	[m]	[kgm^{-2}]	[kgm^{-2}]	[kgm^{-2}]	[mm]	[mm]	[mm]	[%]	[years]	m	f	w	nw	
51.2	1.691	-	-	-	17.9	-	14.0	10.8	3.1	29.0	23.3		f	w		DISTANCE-RUNNING
50.2	1.669	0.905	0.936	17.6	18.0	-0.4	24.0	20.2	3.8	18.6	27.4		f	w		RUNNING
58.0	1.790	0.940	1.024	18.3	18.1	0.2	5.8	4.1	1.7	41.5	18.1	m		w		CYCLING
55.2	1.740	-	-	-	18.2	-	29.8	26.6	3.2	11.9	20.8		f	w		MID-DISTANCE-RUNNING
54.9	1.700	-	-	-	19.0	-	55.0	47.4	7.6	16.1	21.7		f	w		DISTANCE-RUNNING
49.2	1.600	0.860	0.920	19.0	19.2	-0.3	28.1	24.8	3.3	13.4	17.3		f	w		GYM ARTISTIC
57.4	1.728	0.958	0.926	18.4	19.2	-0.8	42.8	36.3	6.5	17.9	27.3		f	w		CYCLING
56.4	1.706	0.913	0.942	19.2	19.4	-0.2	43.1	39.5	3.6	9.0	18.8		f		nw	SOCCER
55.9	1.678	0.833	0.982	21.2	19.9	1.3	17.2	15.3	2.0	12.8	21.9		f	w		ATLETISM
65.6	1.817	0.940	0.993	20.4	19.9	0.5	18.8	13.7	5.2	37.8	19.3	m			nw	SWIMMING
67.6	1.830	0.960	1.020	20.4	20.2	0.2	60.2	53.7	6.5	12.0	28.9		f		nw	SWIMMING
57.5	1.684	0.917	0.937	19.7	20.3	-0.5	38.3	35.7	2.6	7.1	18.9		f		nw	SWIMMING
61.5	1.740	-	-	-	20.3	-	30.8	26.6	4.1	15.5	20.3		f		nw	SWIMMING
70.3	1.855	0.977	0.985	20.6	20.4	0.1	46.4	40.1	6.3	15.7	27.0	m			nw	TENIS
69.2	1.835	-	-	-	20.6	-	8.7	6.5	2.2	33.4	22.8	m		w		TRIATHLON
59.4	1.690	0.900	0.990	20.7	20.8	-0.1	18.5	15.5	3.0	19.3	21.8		f		nw	SWIMMING
66.3	1.784	0.965	0.973	20.4	20.8	-0.4	7.6	6.1	1.5	24.5	18.7	m		w		CYCLING
53.8	1.605	0.878	0.864	20.2	20.9	-0.7	44.1	38.9	5.3	13.5	25.3		f	w		SWIMMING
57.9	1.663	0.872	0.929	21.2	20.9	0.2	48.0	42.8	5.2	12.2	31.8	m		w		LONG-DISTANCE-RUNNING
61.3	1.700	0.980	0.950	19.5	21.2	-1.7	26.1	23.4	2.7	11.5	27.7		f		nw	SWIMMING
66.1	1.760	0.940	-	21.2	21.3	-0.2	6.4	5.2	1.2	23.3	19.7	m		w		GYM ARTISTIC
49.6	1.522	0.814	0.855	21.2	21.4	-0.2	27.9	24.3	3.6	15.0	32.2		f	w		RUNNING
66.8	1.764	0.950	0.980	21.1	21.5	-0.3	46.5	44.0	2.6	5.9	19.8		f		nw	SWIMMING
65.5	1.740	0.910	-	21.9	21.6	0.3	16.7	13.7	3.0	21.8	24.0	m		w		GYM ARTISTIC
60.4	1.663	0.899	0.918	21.4	21.8	-0.4	29.1	25.5	3.6	14.0	34.2		f	w		ROAD RACE CYCLING
63.9	1.710	0.900	-	22.0	21.9	0.2	11.7	9.8	1.9	19.2	22.5	m		w		GYM ARTISTIC
62.2	1.687	0.934	0.873	20.9	21.9	-0.9	58.2	55.2	3.0	5.5	27.0		f		nw	SWIMMING
60.3	1.660	0.890	0.950	21.6	21.9	-0.3	33.1	29.2	3.9	13.3	19.3		f	w		GYM ARTISTIC
72.0	1.810	-	-	-	22.0	-	24.8	19.3	5.5	28.4	25.9	m		w		TRIATHLON
72.3	1.809	0.950	0.978	22.3	22.1	0.2	22.6	18.1	4.4	24.4	22.5	m			nw	JUDO
60.3	1.652	0.882	0.945	21.9	22.1	-0.2	44.9	37.8	7.2	18.9	21.9		f	w		PENTATHLON
70.8	1.790	-	-	-	22.1	-	17.2	14.9	2.3	15.6	19.3	m		w		TRIATHLON
61.7	1.670	0.900	0.930	21.8	22.1	-0.4	44.6	39.4	5.2	13.2	22.2		f	w		GYM ARTISTIC
58.2	1.619	0.860	0.867	22.2	22.2	0.0	51.1	43.1	8.0	18.7	19.3		f	w		SWIMMING
55.3	1.570	0.850	0.860	22.0	22.4	-0.5	36.9	32.6	4.4	13.4	22.4		f	w		GYM ARTISTIC
55.3	1.570	0.849	0.860	22.0	22.4	-0.4	56.2	52.1	4.1	7.8	18.7		f		nw	SWIMMING
60.5	1.642	0.870	0.864	22.4	22.4	0.0	6.3	5.4	1.0	18.3	21.5	m		w		ATLETISM
59.2	1.620	0.899	0.905	21.5	22.6	-1.0	87.4	81.7	5.7	7.0	27.8		f			SWIMMING
68.8	1.745	0.898	0.959	23.3	22.6	0.7	40.9	33.2	7.7	23.2	34.3	m		w		TRIATHLON
76.7	1.840	0.940	1.080	23.5	22.7	0.8	20.8	18.7	2.2	11.7	25.5	m			nw	HOCKEY ICE
69.2	1.744	0.919	0.990	22.9	22.8	0.1	14.2	11.1	3.1	27.7	20.4	m			nw	SOCCER
74.8	1.813	-	-	-	22.8	-	18.6	15.6	3.0	19.5	20.8	m			nw	SWIMMING
70.9	1.763	0.940	0.941	22.7	22.8	-0.1	68.7	62.3	6.4	10.3	20.9		f		nw	TENIS
74.1	1.799	0.941	1.034	23.2	22.9	0.3	9.5	7.8	1.8	22.5	22.6	m		w		CROSS COUNTRY SKIING
68.5	1.723	0.928	0.952	22.7	23.1	-0.4	7.9	5.8	2.1	36.0	32.8	m		w		CYCLING
64.5	1.670	0.901	0.931	22.7	23.1	-0.4	8.8	6.8	2.1	30.4	20.1	m			nw	SOCCER
64.8	1.673	0.950	0.909	21.6	23.2	-1.5	70.7	64.7	6.1	9.4	19.5		f		nw	SOCCER
77.2	1.824	0.944	1.045	23.8	23.2	0.6	80.0	72.5	7.5	10.3	18.1		f		nw	VOLLEYBALL
75.1	1.790	0.950	1.020	23.4	23.4	0.0	7.2	5.8	1.4	23.3	26.8	m		w		SNOWBOARDING
80.8	1.856	0.970	1.026	23.8	23.5	0.3	25.8	19.1	6.7	35.0	32.9	m		w		TRIATHLON
71.3	1.742	0.957	0.967	22.7	23.5	-0.8	48.4	44.1	4.3	9.8	23.8	m			nw	SOCCER
64.9	1.660	0.896	0.839	23.1	23.6	-0.4	76.4	73.3	3.1	4.2	22.8		f		nw	FIELD HOCKEY
83.8	1.882	1.021	1.031	23.1	23.7	-0.5	52.3	45.4	6.9	15.1	21.4	m			nw	SOCCER
83.0	1.870	1.000	1.070	23.5	23.7	-0.2	9.1	6.9	2.3	33.3	26.4	m			nw	SWIMMING
71.5	1.733	-	-	-	23.8	-	83.0	77.2	5.8	7.5	19.5		f		nw	WATERPOLO
77.2	1.800	0.970	1.010	23.4	23.8	-0.4	23.6	21.3	2.4	11.0	28.3	m			nw	HOCKEY ICE
85.0	1.885	-	-	-	23.9	-	11.8	9.6	2.2	23.0	25.0	m		w		TRIATHLON
86.8	1.899	1.008	1.063	24.0	24.1	0.0	50.5	42.8	7.7	18.0	23.8	m		w		CYCLING
70.8	1.710	-	-	-	24.2	-	56.8	51.9	4.8	9.3	20.1		f		nw	WATERPOLO
66.0	1.650	0.870	-	24.4	24.2	0.1	8.7	7.0	1.7	24.5	24.0	m		w		GYM ARTISTIC
69.0	1.683	-	-	-	24.4	-	52.2	47.1	5.2	11.0	24.3		f		nw	KAYAK
90.0	1.920	-	-	-	24.4	-	15.4	13.2	2.2	16.6	23.0	m			nw	SWIMMING
71.6	1.710	0.933	0.954	23.8	24.5	-0.7	80.0	75.8	4.1	5.5	19.3		f		nw	VOLLEYBALL
80.0	1.800	0.960	1.040	24.5	24.7	-0.2	34.8	31.1	3.7	11.9	23.7	m			nw	HOCKEY ICE
67.8	1.657	0.884	0.853	24.5	24.7	-0.2	158.8	149.9	8.9	5.9	20.3		f		nw	BASKETTBALL
71.4	1.700	-	-	-	24.7	-	35.5	29.6	5.9	19.7	25.2	m		w		TRIATHLON
75.6	1.738	0.917	0.913	25.1	25.0	0.1	64.6	55.9	8.7	15.6	29.2		f		nw	TRIATHLON
72.2	1.696	0.920	0.898	24.5	25.1	-0.6	9.1	6.9	2.2	32.3	27.0	m		w		CYCLING
73.4	1.701	-	-	-	25.4	-	91.0	85.9	5.2	6.0	23.7		f		nw	WATERPOLO
90.1	1.877	1.006	1.008	25.3	25.6	-0.3	8.7	6.9	1.8	26.7	24.1	m		w		ATLETISM
74.5	1.705	-	-	-	25.6	-	91.1	81.5	9.5	11.7	20.2		f		nw	KAYAK
85.4	1.796	0.967	0.964	26.1	26.5	-0.4	43.5	38.0	5.6	14.6	22.3	m			nw	SOCCER
79.0	1.727	0.920	0.920	26.4	26.5	-0.1	114.6	107.1	7.5	7.0	21.0		f		nw	BASKETTBALL
91.4	1.840	0.970	1.077	27.1	27.0	0.1	118.1	113.0	5.2	4.6	23.1		f		nw	ROWING OPEN
95.0	1.845	0.982	0.957	27.8	27.9	-0.1	54.7	46.3	8.4	18.0	21.3	m			nw	BASKETTBALL
98.1	1.839	1.000	1.030	28.3	29.0	-0.7	35.9	31.5	4.4	13.9	18.5	m			nw	RUGBY
**68.5**	**1.738**	**0.926**	**0.958**	**22.4**	**22.6**	**-0.2**	**39.9**	**35.5**	**4.3**	**17.0**	**23.4**	**MEAN**				
**11.07**	**0.09**	**0.05**	**0.06**	**2.24**	**2.28**	**0.51**	**29.87**	**28.37**	**2.17**	**8.71**	**4.10**	**SD**				

Table A2

GROUPS		*m*	*h*	*s*	*l*	MI	BMI	D_I	D_E	D_F	$D_{F,\%}$	N
		[kg]	[m]	[m]	[m]	[kgm^{-2}]	[kgm^{-2}]	[mm]	[mm]	[mm]	[%]	
**ALL**	**MEAN**	68.5	1.738	0.926	0.958	22.4	22.6	39.9	35.5	4.3	14.1	76
	**SD**	11.1	0.086	0.046	0.063	2.2	2.3	29.9	28.4	2.2	6.1	
**ALL_m**	**MEAN**	74.2	1.789	0.946	0.991	23.1	23.1	22.6	19.0	3.6	18.0	37
	**SD**	10.0	0.073	0.042	0.052	2.1	2.1	15.9	14.1	2.1	5.4	
**ALL_f**	**MEAN**	63.2	1.689	0.906	0.929	21.7	22.1	56.2	51.2	5.0	10.3	39
	**SD**	9.3	0.067	0.042	0.058	2.2	2.3	30.9	29.7	2.0	4.0	
**ALL_w**	**MEAN**	64.5	1.720	0.908	0.946	21.8	21.7	24.8	21.0	3.8	17.2	36
	**SD**	10.5	0.090	0.048	0.061	1.9	2.0	15.8	13.9	2.2	5.0	
**ALL_nw**	**MEAN**	72.2	1.754	0.942	0.967	23.0	23.4	53.5	48.7	4.8	11.3	40
	**SD**	10.4	0.079	0.037	0.064	2.4	2.2	33.0	31.6	2.1	5.7	
**ALL_m_w**	**MEAN**	70.9	1.769	0.931	0.979	22.6	22.6	17.6	14.4	3.2	19.7	21
	**SD**	8.8	0.076	0.044	0.061	1.8	1.8	14.4	12.4	2.2	4.6	
**ALL_m_nw**	**MEAN**	78.6	1.815	0.963	1.002	23.7	23.8	29.2	25.1	4.1	15.9	16
	**SD**	10.0	0.060	0.033	0.044	2.3	2.4	15.8	14.2	1.9	5.8	
**ALL_f_w**	**MEAN**	55.6	1.651	0.877	0.913	20.7	20.4	34.8	30.1	4.7	13.7	15
	**SD**	4.2	0.059	0.038	0.041	1.6	1.7	12.1	10.4	1.8	3.0	
**ALL_f_nw**	**MEAN**	67.9	1.713	0.925	0.939	22.4	23.1	69.6	64.4	5.2	8.2	24
	**SD**	8.4	0.062	0.033	0.065	2.3	2.1	31.7	30.4	2.1	3.1	
**C1_m_w**	**MEAN**	65.4	1.715	0.905	-	22.4	22.3	10.9	8.9	1.9	18.1	4
	**SD**	1.0	0.048	0.029	-	1.4	1.3	4.4	3.7	0.7	1.5	
**C2_m_w**	**MEAN**	75.1	1.780	0.936	0.964	23.7	23.5	20.5	16.1	4.3	20.3	4
	**SD**	13.0	0.109	0.063	0.073	1.2	1.4	16.2	12.9	3.4	4.4	
**C3_m_w**	**MEAN**	69.1	1.793	0.944	1.026	21.6	21.5	7.5	5.9	1.6	22.2	3
	**SD**	9.6	0.005	0.006	0.007	2.9	2.9	1.9	1.8	0.2	6.2	
**C4_m_w**	**MEAN**	73.7	1.804	-	-	-	22.7	19.6	16.0	3.6	19.2	5
	**SD**	6.4	0.068	-	-	-	1.7	10.8	9.1	1.9	4.5	
**C5_m_w**	**MEAN**	70.3	1.753	0.939	0.963	22.6	22.8	24.6	20.9	3.7	19.3	5
	**SD**	10.6	0.093	0.051	0.062	1.8	1.9	22.5	20.0	2.6	6.4	
**C1_m_nw**	**MEAN**	79.2	1.828	0.968	1.050	23.7	23.7	22.1	19.5	2.6	14.0	4
	**SD**	2.9	0.034	0.025	0.032	0.5	0.8	10.5	10.0	0.7	7.3	
**C2_m_nw**	**MEAN**	75.8	1.832	0.962	0.978	22.8	22.6	35.6	29.6	6.1	19.0	4
	**SD**	13.1	0.022	0.020	0.015	3.5	3.7	17.6	16.1	1.7	6.2	
**C3_m_nw**	**MEAN**	84.7	1.791	0.979	0.999	25.5	26.3	42.1	37.8	4.3	10.6	2
	**SD**	19.0	0.069	0.030	0.045	4.0	3.9	8.9	8.9	0.0	2.3	
**C4_m_nw**	**MEAN**	82.4	1.867	-	-	-	23.6	17.0	14.4	2.6	15.3	2
	**SD**	10.7	0.076	-	-	-	1.2	2.2	1.6	0.6	1.5	
**C5_m_nw**	**MEAN**	75.7	1.773	0.952	0.979	23.7	24.0	29.7	25.3	4.4	17.7	4
	**SD**	10.4	0.089	0.054	0.042	1.6	1.7	21.4	19.2	2.2	5.5	
**C1_f_w**	**MEAN**	56.6	1.625	0.875	0.915	21.1	21.4	35.7	31.5	4.2	11.7	4
	**SD**	5.7	0.048	0.024	0.039	1.4	1.5	6.9	6.1	0.8	0.1	
**C2_f_w**	**MEAN**	57.1	1.639	0.863	0.915	21.4	21.3	39.3	33.7	5.6	13.7	4
	**SD**	2.8	0.033	0.022	0.059	0.9	1.1	15.1	12.5	2.7	2.4	
**C3_f_w**	**MEAN**	60.4	1.663	0.899	0.918	21.4	21.8	29.1	25.5	3.6	12.3	1
	**SD**	-	-	-	-	-	-	-	-	-	-	
**C4_f_w**	**MEAN**	53.8	1.710	-	-	-	18.4	32.9	28.3	4.7	15.7	3
	**SD**	2.2	0.026	-	-	-	0.6	20.7	18.3	2.6	6.1	
**C5_f_w**	**MEAN**	52.4	1.640	0.892	0.906	19.1	19.6	31.6	26.9	4.6	14.6	3
	**SD**	4.3	0.106	0.073	0.044	1.9	1.7	9.9	8.4	1.6	1.4	
**C1_f_nw**	**MEAN**	60.9	1.698	0.922	0.955	20.6	21.2	40.2	36.2	4.0	11.1	4
	**SD**	5.1	0.106	0.060	0.070	1.0	1.0	21.0	19.6	1.7	3.7	
**C2_f_nw**	**MEAN**	73.3	1.721	0.915	0.907	24.7	24.8	101.7	93.8	7.9	8.7	4
	**SD**	5.0	0.045	0.023	0.038	1.5	1.5	44.3	43.8	1.2	3.6	
**C3_f_nw**	**MEAN**	67.0	1.709	0.928	0.935	22.3	22.8	70.8	67.1	3.7	5.4	6
	**SD**	12.4	0.080	0.029	0.085	2.6	2.3	29.5	28.3	1.4	1.1	
**C4_f_nw**	**MEAN**	70.1	1.712	-	-	-	23.9	67.5	61.7	5.8	9.2	6
	**SD**	4.6	0.021	-	-	-	1.9	24.7	23.5	1.9	2.8	
**C5_f_nw**	**MEAN**	67.5	1.728	0.935	0.963	22.1	22.6	68.4	63.1	5.3	7.9	4
	**SD**	9.0	0.066	0.016	0.058	2.2	2.2	17.5	16.4	1.8	1.8	

Table A3: Thickness value differences ABS(δ_I) at the individual sites

Absolute values of differences of the three measurers from their mean values for thickness measurements with fibrous structures included (index: "I"). For the expert group (C1 and C2), number of comparisons at each of the eight sites is: N=(16+16)3=96; for novices (C3-C5): N=(12+16+16)3=132. Abbreviations: UA: upper abdomen, LA: lower abdomen, EO: external oblique, DT: distal triceps, BR: brachioradialis, LT: lateral thigh, FT: front thigh, and MC: medial calf. IQR: inter quartile range, Q3: third quartile (First 75% of values when ordered according to increasing values). Data correspond to Figs. 6a and 6b.

C1, C2	UA	LA	EO	ES	DT	BR	FT	MC
MEDIAN [mm]	0.15	0.19	0.14	0.11	0.15	0.06	0.10	0.11
IQR [mm]	0.22	0.28	0.24	0.17	0.22	0.08	0.14	0.12
Q3 [mm]	0.29	0.36	0.29	0.23	0.31	0.11	0.17	0.19
C3 - C5	UA	LA	EO	ES	DT	BR	FT	MC
MEDIAN [mm]	0.24	0.47	0.24	0.22	0.22	0.14	0.18	0.19
IQR [mm]	0.37	0.72	0.35	0.27	0.37	0.28	0.30	0.27
Q3 [mm]	0.47	0.88	0.48	0.36	0.45	0.34	0.37	0.35

Table A4: Thickness value differences ABS(δ_E) at the individual sites

Absolute values of differences of the three measurers from their mean values for thickness measurements with fibrous structures excluded (index: "E"). For number of comparisons and abbreviations see Table A3. Data correspond to Figs. 6c and 6d.

C1, C2	UA	LA	EO	ES	DT	BR	FT	MC
MEDIAN [mm]	0.19	0.19	0.12	0.12	0.16	0.08	0.15	0.13
IQR [mm]	0.24	0.51	0.22	0.15	0.22	0.10	0.19	0.19
Q3 [mm]	0.31	0.59	0.28	0.20	0.28	0.13	0.25	0.26
C3 - C5	UA	LA	EO	ES	DT	BR	FT	MC
MEDIAN [mm]	0.23	0.51	0.21	0.20	0.22	0.15	0.31	0.22
IQR [mm]	0.41	0.93	0.40	0.24	0.36	0.28	0.39	0.30
Q3 [mm]	0.50	1.10	0.49	0.34	0.47	0.35	0.52	0.40

Table A5: Relative thickness value differences at the individual sites related to the given subcutaneous adipose tissue thickness (d_I)

ABS($\delta_{I,rel}$)=100·ABS(δ_I)/d_I. The table shows the percentages of measurement differences from the three measurers' mean for thickness measurements with fibrous structures included (index: "I"). For number of comparisons and abbreviations see Table A3. Data correspond to Figs. A1a and b.

C1, C2	UA	LA	EO	ES	DT	BR	FT	MC
MEDIAN [%]	4.61	2.62	6.10	4.35	5.33	4.15	2.12	3.41
IQR [%]	6.64	3.26	12.16	5.87	6.77	10.22	3.07	5.43
Q3 [%]	8.69	4.38	14.84	8.00	8.69	12.24	3.94	7.57
C3 - C5	UA	LA	EO	ES	DT	BR	FT	MC
MEDIAN [%]	6.53	5.53	12.65	5.99	6.94	10.39	4.27	5.63
IQR [%]	9.44	6.86	21.58	10.93	10.49	17.35	7.26	14.68
Q3 [%]	12.59	9.53	27.36	13.57	12.56	22.05	8.84	17.00

Table A6: Relative thickness value differences at the individual sites related to the given subcutaneous adipose tissue thickness (d_E)
$ABS(\delta_{E,rel})=100 \cdot ABS(\delta_E)/d_E$. The table shows the percentages of measurement differences from the three measurers' mean for thickness measurements with fibrous structures included (index: "I"). For number of comparisons and for abbreviations see Table A3. Data correspond to Figs. A1c and A1b.

C1, C2	UA	LA	EO	ES	DT	BR	FT	MC
MEDIAN [%]	4.80	4.21	7.04	4.45	5.39	6.20	3.91	5.94
IQR [%]	9.51	5.93	12.37	4.96	8.59	9.23	4.60	8.92
Q3 [%]	11.96	7.88	15.16	7.20	10.20	12.05	6.17	11.87
C3 - C5	UA	LA	EO	ES	DT	BR	FT	MC
MEDIAN [%]	8.42	9.13	16.02	6.71	7.76	12.39	6.88	8.54
IQR [%]	11.64	9.60	21.98	8.31	14.60	16.81	8.00	15.79
Q3 [%]	15.13	13.87	28.56	11.47	17.84	22.68	11.50	18.94

Fig. A1: Percentages of measurement differences δ_{rel} with respect to the subcutaneous adipose tissue thicknesses (d) at the individual sites.
According data are presented in Tables A5 and A6. Abbreviations. UA: upper abdomen, LA: lower abdomen, EO: external oblique, DT: distal triceps, BR: brachioradialis, LT: lateral thigh, FT: front thigh, and MC: medial calf.
Index "I": fibrous structures (fasciae) included, index "E": fibrous structures excluded.
A1a: Experience examiners (C1,C2): $ABS(\delta_{I,rel})=100 \cdot ABS(\delta_I)/d_I$.
Number of comparisons at each of the eight sites is: N=(16+16)3=96.
A1b: Novices (C3-5): $ABS(\delta_{I,rel})=100 \cdot ABS(\delta_I)/d_I$.
Number of comparisons at each of the eight sites is: N=(12+16+16)3=132.
A1c: Experience examiners: ABS (δ_E) for each of the eight sites.
$ABS(\delta_{E,rel})=100 \cdot ABS(\delta_E)/d_E$. N=96.
A1d: Novices: ABS (δ_E) for each of the eight sites.
$ABS(\delta_{E,rel})=100 \cdot ABS(\delta_E)/d_E$. N=132.

A1a:

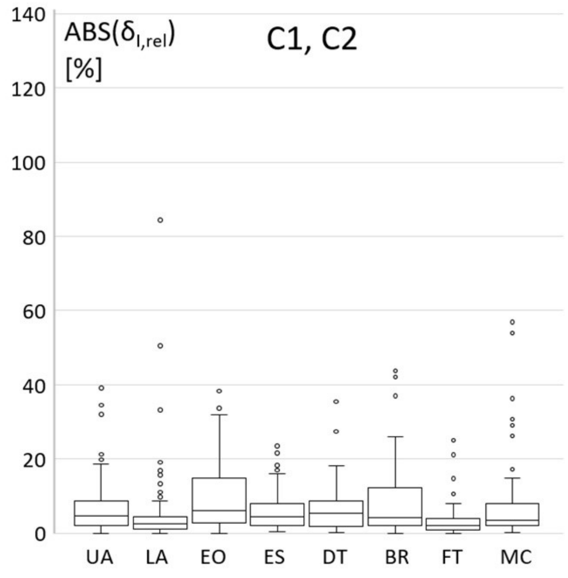

A1b:

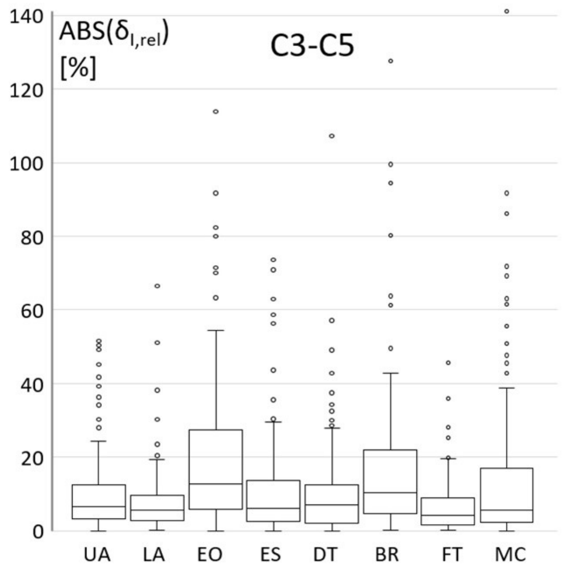

A1c:

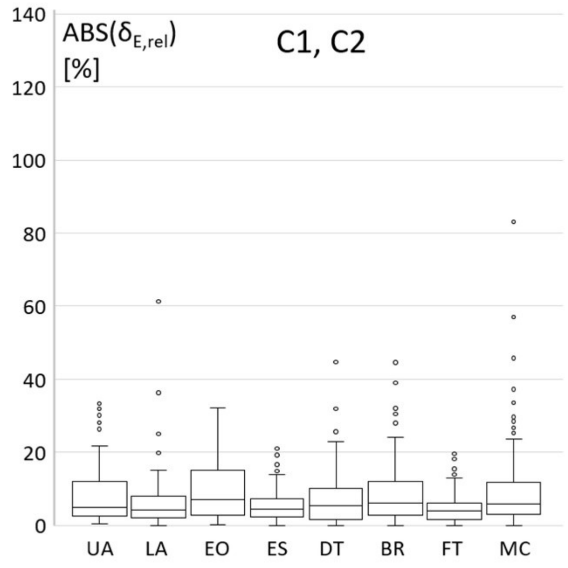

A1d:

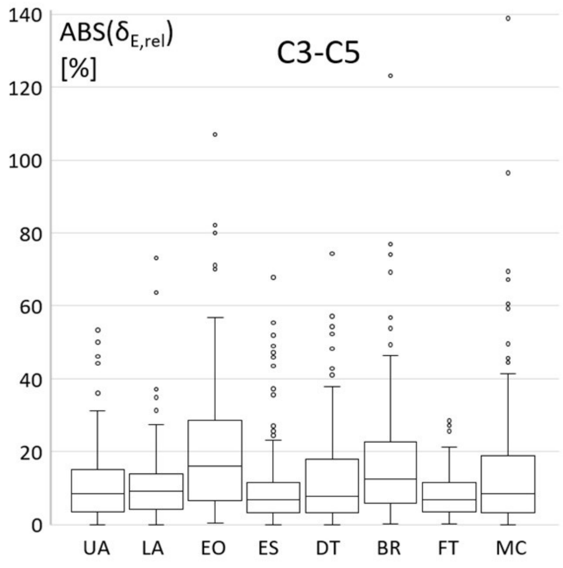

Table A7: Preliminary normative data for the subcutaneous adipose tissue (SAT) sums of the eight standardised sites [12].

Sum of SAT (mm)	Valuation	Comment
**Competitive Athletes - Female**		
below 25	Extremely low	Medical surveillance recommended
25 to 35	Very low	Surveillance recommended
35 to 50	Low	Desirable range
50 to 70		Noticeable ballast weight
above 70		Considerable ballast weight
**Competitive Athletes - Male**		
below 12	Extremely low	Medical surveillance recommended
12 to 20	Very low	Surveillance recommended
20 to 30	Low	Desirable range
30 to 50		Noticeable ballast weight
above 50		Considerable ballast weight
**General Public - Female**		
below 25	Extremely low	Medical surveillance recommended
25 to 35	Very low	Surveillance recommended
35 to 80	Low	Desirable range
80 to 110		Noticeable ballast weight
110 to 140		Considerable ballast weight
140 to 180	High	Surveillance recommended
above 180	Very high	Medical surveillance recommended
**General Public - Male**		
below 12	Extremely low	Medical surveillance recommended
12 to 20	Very low	Surveillance recommended
20 to 60	Low	Desirable range
60 to 100		Noticeable ballast weight
100 to 130		Considerable ballast weight
130 to 180	High	Surveillance recommended
above 180	Very high	Medical surveillance recommended

Table A8: D_I and D_E in mm of all 76 athletes. M1, M2, M3: measurers of each centre

N	CENTRE	SUBJECT	$D_{I,M1}$	$D_{I,M2}$	$D_{I,M3}$	$D_{I,mean}$	$D_{E,M1}$	$D_{E,M2}$	$D_{E,M3}$	$D_{E,mean}$
1	C1	S1	37.40	36.83	36.55	36.93	32.20	33.16	32.37	32.58
2	C1	S2	44.39	44.70	44.68	44.59	39.07	39.04	40.06	39.39
3	C1	S3	27.65	28.66	28.08	28.13	24.96	25.56	23.92	24.81
4	C1	S4	31.96	32.46	34.79	33.07	28.28	28.66	30.67	29.20
5	C1	S5	6.40	6.05	6.59	6.35	4.78	4.94	5.74	5.15
6	C1	S6	16.35	16.44	17.17	16.65	13.64	13.64	13.72	13.67
7	C1	S7	8.45	8.96	8.65	8.69	7.10	6.75	7.08	6.98
8	C1	S8	11.81	12.01	11.36	11.73	10.11	9.87	9.54	9.84
9	C1	S9	55.86	56.69	55.93	56.16	51.72	53.30	51.25	52.09
10	C1	S10	35.08	34.73	34.51	34.77	30.81	30.21	32.23	31.08
11	C1	S11	20.93	20.55	21.04	20.84	19.02	18.34	18.63	18.66
12	C1	S12	23.15	23.86	23.87	23.63	21.80	21.42	20.63	21.28
13	C1	S13	8.96	8.93	9.51	9.13	6.80	6.60	7.16	6.85
14	C1	S14	18.16	17.87	19.36	18.46	15.10	15.24	16.09	15.48
15	C1	S15	61.91	58.04	60.61	60.19	55.09	52.05	54.09	53.74
16	C1	S16	25.74	26.83	25.71	26.09	22.98	23.66	23.55	23.40
17	C2	S1	26.32	26.05	25.13	25.83	20.35	19.59	17.48	19.14
18	C2	S2	40.60	41.22	40.96	40.93	33.91	32.75	32.97	33.21
19	C2	S3	8.57	8.30	9.31	8.73	6.87	6.62	7.17	6.89
20	C2	S4	44.58	43.50	44.28	44.12	39.91	38.84	37.85	38.87
21	C2	S5	45.73	44.55	44.55	44.94	38.83	38.35	36.20	37.79
22	C2	S6	16.83	17.39	17.49	17.24	15.52	15.41	14.90	15.28
23	C2	S7	6.28	6.32	6.41	6.34	5.56	5.25	5.25	5.35
24	C2	S8	51.28	51.15	50.88	51.10	42.82	43.46	42.90	43.06
25	C2	S9	18.44	19.72	18.35	18.84	13.43	14.89	12.67	13.66
26	C2	S10	22.51	22.42	22.80	22.58	17.56	18.71	18.17	18.15
27	C2	S11	54.94	55.00	54.02	54.65	46.82	46.21	45.87	46.30
28	C2	S12	112.41	116.50	107.55	112.15	101.68	110.46	102.20	104.78
29	C2	S13	63.77	64.66	65.31	64.58	57.64	57.88	52.04	55.85
30	C2	S14	44.78	46.56	47.88	46.41	38.22	40.38	41.71	40.10
31	C2	S15	159.35	160.77	156.15	158.76	149.70	153.28	146.63	149.87
32	C2	S16	67.95	70.18	68.06	68.73	60.31	64.22	62.47	62.33
33	C3	S1	9.14	11.29	8.18	9.54	6.94	9.46	6.98	7.79
34	C3	S2	6.15	7.65	7.76	7.19	5.39	5.88	6.22	5.83
35	C3	S3	30.14	28.58	28.57	29.10	25.97	26.49	24.14	25.53
36	C3	S4	5.75	5.94	5.70	5.80	3.58	4.43	4.29	4.10
37	C3	S5	113.71	125.21	115.42	118.11	106.60	120.84	111.43	112.96
38	C3	S6	59.72	55.74	59.19	58.22	58.16	50.27	57.18	55.20
39	C3	S7	87.06	84.18	90.85	87.36	81.59	79.00	84.43	81.67
40	C3	S8	38.09	36.88	39.90	38.29	34.77	34.45	37.99	35.74
41	C3	S9	80.03	69.54	79.62	76.40	76.68	66.06	77.29	73.34
42	C3	S10	47.30	45.63	46.70	46.54	44.42	43.18	44.25	43.95
43	C3	S11	46.94	49.86	48.39	48.40	43.39	45.11	43.77	44.09
44	C3	S12	35.43	38.71	33.45	35.86	29.49	34.59	30.37	31.48
45	C4	S1	17.94	18.49	15.26	17.23	14.58	17.35	12.77	14.90
46	C4	S2	13.38	12.21	9.69	11.76	10.99	10.55	7.13	9.56
47	C4	S3	25.78	23.20	25.48	24.82	18.00	17.69	22.29	19.33
48	C4	S4	8.97	8.18	8.98	8.71	6.98	6.12	6.48	6.53
49	C4	S5	29.81	31.02	28.58	29.80	26.75	28.84	24.28	26.62
50	C4	S6	58.59	54.44	51.93	54.99	49.38	49.04	43.63	47.35
51	C4	S7	32.97	36.65	36.86	35.49	25.97	31.59	31.37	29.64
52	C4	S8	13.60	14.67	13.64	13.97	10.44	11.85	10.19	10.83
53	C4	S9	28.33	32.42	31.51	30.75	24.45	28.56	26.87	26.63
54	C4	S10	15.20	15.40	15.63	15.41	12.28	14.19	13.20	13.22
55	C4	S11	19.53	19.37	16.86	18.59	16.24	16.13	14.29	15.55
56	C4	S12	97.95	82.32	92.89	91.05	89.65	72.66	82.26	81.52
57	C4	S13	55.85	51.55	49.33	52.24	52.79	45.11	43.26	47.05
58	C4	S14	55.69	54.98	59.58	56.75	47.95	50.37	57.46	51.93
59	C4	S15	82.47	83.22	83.20	82.96	75.45	76.40	79.73	77.19
60	C4	S16	90.95	89.35	92.71	91.00	86.50	82.95	88.09	85.85
61	C5	S1	49.74	45.31	48.96	48.00	44.92	39.85	43.59	42.79
62	C5	S2	43.19	39.72	45.39	42.77	36.31	33.68	38.89	36.29
63	C5	S3	30.61	25.22	27.97	27.93	27.49	21.83	23.56	24.29
64	C5	S4	25.57	22.04	24.36	23.99	22.63	17.34	20.71	20.23
65	C5	S5	8.30	7.33	7.24	7.62	6.62	5.97	5.78	6.12
66	C5	S6	53.85	46.70	51.02	50.52	47.88	37.69	42.93	42.83
67	C5	S7	7.98	8.71	7.00	7.90	5.67	6.22	5.53	5.81
68	C5	S8	8.22	9.91	9.15	9.09	6.98	6.20	7.44	6.87
69	C5	S9	80.62	76.31	83.00	79.98	77.19	71.79	78.55	75.84
70	C5	S10	71.87	66.91	73.37	70.72	65.19	60.26	68.51	64.65
71	C5	S11	45.05	40.25	45.26	43.52	39.73	34.51	39.65	37.96
72	C5	S12	43.68	41.04	44.43	43.05	41.38	36.74	40.31	39.48
73	C5	S13	52.05	50.47	54.37	52.30	47.00	42.22	47.05	45.42
74	C5	S14	81.56	78.33	80.11	80.00	73.58	70.64	73.33	72.52
75	C5	S15	10.26	7.43	8.72	8.80	7.11	6.04	7.09	6.75
76	C5	S16	15.05	12.67	14.80	14.17	12.77	9.63	10.90	11.10
